# Supplementary material for: Effect of HIV status and antiretroviral treatment on treatment outcomes of tuberculosis patients in a rural primary healthcare clinic in South Africa
Source: PLoS One. 2022 Oct 12;17(10):e0274549. doi: 10.1371/journal.pone.0274549 (PMC9555649; doi:10.1371/journal.pone.0274549)
Supplement: Attachment — Submitted filename: PONE-D-21-05746_reviewer.pdf [file pone.0274549.s002.pdf]

PLOS ONE

Effect of HIV status and antiretroviral treatment on treatment outcomes of tuberculosis patients in a rural primary healthcare clinic in South Africa

--Manuscript Draft--

**Manuscript Number:**	PONE-D-21-05746
**Article Type:**	Research Article
**Full Title:**	Effect of HIV status and antiretroviral treatment on treatment outcomes of tuberculosis patients in a rural primary healthcare clinic in South Africa
**Short Title:**	HIV Infection and treatment outcomes among Tuberculosis patients in rural South Africa
**Corresponding Author:**	Peter S Nyasulu, PhD<br>Stellenbosch University Faculty of Medicine and Health Sciences<br>Cape Town, Western Cape SOUTH AFRICA
**Keywords:**	TB; Treatment outcomes; co-infection; HIV Status, KwaZulu Natal province, South Africa
**Abstract:**	Background<br><br>Tuberculosis (TB) remains the leading cause of death among human immunodeficiency virus (HIV) infected individuals in South Africa. Despite the implementation of HIV/TB integration services at primary healthcare facility level, the effect of HIV on TB treatment outcomes has not been well investigated. To provide evidence base for TB treatment outcome improvement to meet End TB Strategy goal, we assessed the effect of HIV status on treatment outcomes of TB patients at a rural clinic in the Ugu Health District, South Africa.<br><br>Methods<br><br>We reviewed medical records involving a cohort of 508 TB patients registered for treatment between 1 January 2013 and 31 December 2015 at rural public sector clinic in KwaZulu-Natal province, South Africa. Data were extracted from National TB Programme clinic cards and the TB case registers routinely maintained at study sites. The effect of HIV status on TB treatment outcomes was determined by using multinomial logistic regression. Estimates used were relative risk ratio (RRR) at 95% confidence intervals (95%CI).<br><br>Results<br><br>A total of 506 patients were included in the analysis. Majority of the patients (88%) were new TB cases, 70% had pulmonary TB and 59% were co-infected with HIV. Most of HIV positive patients were on antiretroviral therapy (ART) (90% (n=268)). About 82% had successful treatment outcome (cured 39.1% (n=198) and completed treatment (42.9% (n=217)), 7% (n=39) died 0.6% (n=3) failed treatment, 3.9% (n=20) defaulted treatment and the rest (6.6% (n=33)) were transferred out of the facility. Using completed treatment as reference, HIV positive patients not on ART relative to negative patients were more likely to have unsuccessful outcomes [RRR, 5.41; 95%CI, 2.11–13.86].<br><br>Conclusions<br><br>The cured rate is below district and provincial targets at this facility. Despite a high ART coverage, HIV status is a strong predictor of the unfavourable TB treatment outcomes.
**Order of Authors:**	Peter S Nyasulu, PhD
	Emery L Ngasama
	Jacques Lukenze Tamuzi
	Lovemore N Sigwadi

Powered by Editorial Manager® and ProduXion Manager® from Aries Systems Corporation

	Lovelyn U Ozougwu
	Ruvimbo BC Nhandara
	Birhanu T Ayele
	Teye Umanah
	Jabulani Ncayiyana
**Additional Information:**	
**Question**	**Response**
**Financial Disclosure**<br><br>Enter a financial disclosure statement that describes the sources of funding for the work included in this submission. Review the submission guidelines for detailed requirements. View published research articles from *PLOS ONE* for specific examples.<br><br>This statement is required for submission and **will appear in the published article** if the submission is accepted. Please make sure it is accurate.<br><br>**Unfunded studies**<br>Enter: *The author(s) received no specific funding for this work.*<br><br>**Funded studies**<br>Enter a statement with the following details:<br>• Initials of the authors who received each award<br>• Grant numbers awarded to each author<br>• The full name of each funder<br>• URL of each funder website<br>• Did the sponsors or funders play any role in the study design, data collection and analysis, decision to publish, or preparation of the manuscript?<br>• **NO** - Include this sentence at the end of your statement: *The funders had no role in study design, data collection and analysis, decision to publish, or preparation of the manuscript.*<br>• **YES** - Specify the role(s) played.<br><br>* typeset	N/A
**Competing Interests**<br><br>Use the instructions below to enter a competing interest statement for this submission. On behalf of all authors,	The authors have declared that no competing interests exist.

disclose any competing interests that could be perceived to bias this work—acknowledging all financial support and any other relevant financial or non-financial competing interests.<br><br>This statement is required for submission and **will appear in the published article** if the submission is accepted. Please make sure it is accurate and that any funding sources listed in your Funding Information later in the submission form are also declared in your Financial Disclosure statement.<br><br>View published research articles from *PLOS ONE* for specific examples.<br><br>**NO authors have competing interests**<br><br>Enter: *The authors have declared that no competing interests exist.*<br><br>**Authors with competing interests**<br><br>Enter competing interest details beginning with this statement:<br><br>*I have read the journal's policy and the authors of this manuscript have the following competing interests: [insert competing interests here]*<br><br>* typeset	
**Ethics Statement**<br><br>Enter an ethics statement for this submission. This statement is required if the study involved:<br><br>• Human participants<br>• Human specimens or tissue<br>• Vertebrate animals or cephalopods<br>• Vertebrate embryos or tissues<br>• Field research<br><br>Write "N/A" if the submission does not require an ethics statement.	The University of the Witwatersrand Human Research Ethics Committee approved the study. Authority to access medical records was obtained from the Director of Health, Ugu District. No attempt was made to link data to the individual identifier, and data was analysed anonymously.

Powered by Editorial Manager® and ProduXion Manager® from Aries Systems Corporation

General guidance is provided below. Consult the submission guidelines for detailed instructions. **Make sure that all information entered here is included in the Methods section of the manuscript.**<br><br>**Format for specific study types**<br><br>**Human Subject Research (involving human participants and/or tissue)**<br>• Give the name of the institutional review board or ethics committee that approved the study<br>• Include the approval number and/or a statement indicating approval of this research<br>• Indicate the form of consent obtained (written/oral) or the reason that consent was not obtained (e.g. the data were analyzed anonymously)<br><br>**Animal Research (involving vertebrate animals, embryos or tissues)**<br>• Provide the name of the Institutional Animal Care and Use Committee (IACUC) or other relevant ethics board that reviewed the study protocol, and indicate whether they approved this research or granted a formal waiver of ethical approval<br>• Include an approval number if one was obtained<br>• If the study involved *non-human primates*, add *additional details* about animal welfare and steps taken to ameliorate suffering<br>• If anesthesia, euthanasia, or any kind of animal sacrifice is part of the study, include briefly which substances and/or methods were applied<br><br>**Field Research**<br><br>Include the following details if this study involves the collection of plant, animal, or other materials from a natural setting:<br>• Field permit number<br>• Name of the institution or relevant body that granted permission	
**Data Availability**<br><br>Authors are required to make all data	Yes - all data are fully available without restriction

Powered by Editorial Manager® and ProduXion Manager® from Aries Systems Corporation

underlying the findings described fully available, without restriction, and from the time of publication. PLOS allows rare exceptions to address legal and ethical concerns. See the PLOS Data Policy and FAQ for detailed information.<br><br>A Data Availability Statement describing where the data can be found is required at submission. Your answers to this question constitute the Data Availability Statement and **will be published in the article**, if accepted.<br><br>**Important:** Stating 'data available on request from the author' is not sufficient. If your data are only available upon request, select 'No' for the first question and explain your exceptional situation in the text box.<br><br>Do the authors confirm that all data underlying the findings described in their manuscript are fully available without restriction?	
**Describe where the data may be found in full sentences. If you are copying our sample text, replace any instances of XXX with the appropriate details.**<br><br>• If the data are **held or will be held in a public repository**, include URLs, accession numbers or DOIs. If this information will only be available after acceptance, indicate this by ticking the box below. For example: *All XXX files are available from the XXX database (accession number(s) XXX, XXX.*).<br>• If the data are all contained **within the manuscript and/or Supporting Information files**, enter the following: *All relevant data are within the manuscript and its Supporting Information files.*<br>• If neither of these applies but you are able to provide **details of access elsewhere**, with or without limitations, please do so. For example:<br><br>*Data cannot be shared publicly because of [XXX]. Data are available from the*	All relevant data are within the manuscript and its Supporting Information files.

Powered by Editorial Manager® and ProduXion Manager® from Aries Systems Corporation

XXX Institutional Data Access / Ethics Committee (contact via XXX) for researchers who meet the criteria for access to confidential data.<br><br>*The data underlying the results presented in the study are available from (include the name of the third party and contact information or URL).*<br>• This text is appropriate if the data are owned by a third party and authors do not have permission to share the data.<br><br>* typeset	
Additional data availability information:	

Powered by Editorial Manager® and ProduXion Manager® from Aries Systems Corporation

Manuscript Click here to access/download;Manuscript;HIV_TB_co-infection_Ugu district 19 February 2021.docx

Effect of HIV status and antiretroviral treatment on treatment outcomes of tuberculosis patients in a rural primary healthcare clinic in South Africa

Peter S Nyasulu[1, 2], Emery Ngasama[2], Jacques L Tamuzi[1], Lovemore N Sigwadhi[1], Lovelyn U Ozougwu[3], Ruvimbo BC Nhandara[2], Birhanu T Ayele[1], Teye Umanah[4] Jabulani Ncayiyana[5]

Affiliations

[1]Division of Epidemiology and Biostatistics, Department of Global Health, Faculty of Medicine and Health Sciences, Stellenbosch University, Cape Town, South Africa

[2]Division of Epidemiology & Biostatistics, School of Public Health, Faculty of Health Sciences, University of the Witwatersrand, Johannesburg, South Africa

[3]Division of Public Health Surveillance and Response, National Institute for Communicable Diseases, Johannesburg, South Africa.

[4]Neuroscience Institute, Hackensack Meridian University at JFK Medical Center, New Jersey, United States of America.

[5]Public Health Medicine, School of Nursing and Public Health, University of KwaZulu-Natal, Durban, South Africa

Abstract

Background: Tuberculosis (TB) remains the leading cause of death among human immunodeficiency virus (HIV) infected individuals in South Africa. Despite the implementation of HIV/TB integration services at primary healthcare facility level, the effect of HIV on TB treatment outcomes has not been well investigated. To provide evidence base for TB treatment outcome improvement to meet End TB Strategy goal, we assessed the effect of HIV status on treatment outcomes of TB patients at a rural clinic in the Ugu Health District, South Africa.

Methods: We reviewed medical records involving a cohort of 508 TB patients registered for treatment between 1 January 2013 and 31 December 2015 at rural public sector clinic in KwaZulu-Natal province, South Africa. Data were extracted from National TB Programme clinic cards and the TB case registers routinely maintained at study sites. The effect of HIV status on TB treatment outcomes was determined by using multinomial logistic regression. Estimates used were relative risk ratio (RRR) at 95% confidence intervals (95%CI).

Results: A total of 506 patients were included in the analysis. Majority of the patients (88%) were new TB cases, 70% had pulmonary TB and 59% were co-infected with HIV. Most of HIV positive patients were on antiretroviral therapy (ART) (90% (n=268)). About 82% had successful treatment outcome (cured 39.1% (n=198) and completed treatment (42.9% (n=217)), 7% (n=39) died 0.6% (n=3) failed treatment, 3.9% (n=20) defaulted treatment and the rest (6.6% (n=33)) were transferred out of the facility. Using completed treatment as reference, HIV positive patients not on ART relative to

negative patients were more likely to have unsuccessful outcomes [RRR, 5.41; 95%CI, 2.11–13.86].

Conclusions: The cured rate is below district and provincial targets at this facility Despite a high ART coverage, HIV status is a strong predictor of the unfavourable TB treatment outcomes.

Key words: TB; Treatment outcomes; co-infection; HIV Status, KwaZulu Natal province, South Africa.

1. Background

Tuberculosis (TB) is one of the major global infectious diseases with a high morbidity and mortality rate. Globally, an estimated 10.0 million (range 8.9–11.0 million) people fell ill with TB in 2019 [1]. There were 1.2 million (range, 1.1–1.3 million) TB deaths among HIV-negative people and an additional 208 000 (range, 177 000–242 000) TB deaths among HIV-positive people [1]. Ninety percent (90%) of all TB cases occur in adults with a high prevalence in males compared to females [2]. Between 2000 and 2017, TB death among HIV-negative people decreased by 29 percent from 1.8 million to 1.3 million worldwide [3] and the increase in incidence is also attributed to the development of multidrug resistant (MDR) and extremely drug resistant (XDR) strains of Mycobacterium tuberculosis [4]. In fact, the human immunodeficiency virus alters the natural history of TB; therefore, people living with HIV (PLWHIV) is 26 times (24–28) more likely to develop active TB disease than those without HIV, amplifying the spread of TB [5]. TB is the most common opportunistic infection and cause of death among human immunodeficiency virus (HIV) infected patients in resource-limited countries [6-7]. In the post-mortem period, the overall prevalence of TB was enormous and accounted for almost 40% of HIV-related facility-based deaths in resource-limited countries [8-9].

HIV prevalence in sub-Saharan Africa has significantly contributed to an increase in the incidence of TB [4]. An estimated 10 million people have been living with TB and 1.5 million have died in 2018 and the global burden of TB falls on 20 low-and middle-income (LMIC) countries, including sub-Saharan Africa [10]. The burden is attributed to the high rate of HIV/AIDS pandemics in those countries. Africa accounted for 84% of all TB/HIV deaths [10]. South Africa ranked fifth among the highest TB incidences

worldwide and first among TB/HIV co-infection cases with more than 65 percent of patients co-infected with TB/HIV [4].

TB accounted for the third highest number of deaths in 2014 (5.4%; n = 33 375), and combined TB and HIV accounted for 35.6% of all-cause mortality in South Africa [5, 11-12]. The total number of people living with HIV in South Africa increased from an estimated 4.64 million in 2002 to 7.97 million by 2019 [13]. There are significant geographical variations in the rate of TB notification in South Africa which are not clearly correlated with the prevalence of HIV at the district level [14-16]. KwaZulu-Natal (KZN) carries the largest burden of HIV and related infections in South Africa (SA), with HIV–TB co-infection estimated at approximately 70% [5, 17].

According to a recent systematic review, ART may be associated with a reduced risk of HIV-associated TB disease in HIV-positive individuals due to a decrease in their viral load and an improvement in their immune system function [18]. On the one hand, while ART reduces new HIV infections, on the other hand, the marked decline in HIV-associated mortality has led to an increase in HIV prevalence and an increase in the number of life-years at TB risk. It is also plausible that HIV-positive people with TB infection may increase with CD4+ T-cell ART recovery, although the available data do not support this [16, 18-19]. In addition, early initiation of ART during TB treatment (within 2–4 weeks) increased AIDS-free survival by 34–68% among patients with advanced HIV disease [7, 20-22]. Despite the inclusion of TB-HIV in the international and South African guidelines, the 2018 mortality rate for co-infected TB-HIV patients in South Africa was 73 (51-99) per 100,000 populations, which is more than three times higher than that for HIV-negative TB patients with a mortality rate of 37 (35–39) per 100,000 population [23]

A recent study in KZN revealed that staff rotation challenges across different services have been attributed to skills deficiency (80 per cent); staff shortage (50 per cent); and high staff turnover, absenteeism and personal preferences (30 per cent) in KZN [5]. Despite a substantial investment in the clinical expansion program in South Africa, sparsely populated rural areas are still constrained in geographic access (seen at a lower PHC utilization rate) and this is a major challenge in provision HIV and TB services in rural areas [24]. In this context, integrated services can promote equity as they maximize the potential benefit for each facility visit for health care [24].

The End TB Strategy aims to reduce TB deaths by 90% and TB incidence rates by 80% in 2030 compared to 2015 [3]. Examining the various challenges that may impact on TB outcomes in rural primary health care in South Africa, the TB elimination target set for 2050 could be compromised if this dual burden of TB and HIV diseases is not controlled [5, 25]. The rate of TB incident stabilizes at a rate higher than that of the general population. These data highlight the need for more research into strategies for finding active cases in rural settings and the need to focus on strengthening primary health care [26]. This is why; this retrospective study has been undertaken to evaluate TB outcomes in HIV positive patients in rural primary healthcare in South Africa

2. Methods

2.1. Study design and population

This retrospective cohort study of TB patients initiated on TB treatment at the Elim clinic, Ugu district was conducted from 1 January 2013 to 31 December 2015. The Ugu district is located in the rural public sector clinic in KwaZulu-Natal (Figure 1). All patients diagnosed with TB irrespective of their age and HIV status were included in the study. Further, we registered patients that were in the TB register in 2012 and died in 2013. We also included as well as those that died or survived during the treatment period. Patients with unknown outcome were excluded from the study.

2.2. Sample size

A total of 478 cases were estimated to be adequate sample that would have the power to detect a significant difference in mortality between HIV positive and negative TB patients on anti-tuberculosis treatment. We used a 95% precision of estimate with power of 80% and estimated risk difference of 6% in outcome between exposed and unexposed.

2.3. Participant selection and definitions

All TB patients of all ages recorded in the TB treatment register, with HIV positive status and started on treatment were included in this study. These were all TB patients on treatment attending care at Elim Clinic, in Ugu District, KZN province within the stipulated time. At admission, patients were screened for other comorbidities including HIV infection. After following the treatment, the outcomes of treatment were recorded in seven mutually exclusive categories. Patients were classified as cured, completed treatment, interrupted treatment, moved, transferred out, failed and died. The main interest of this study was to compare the likelihood of observing two main outcomes.

These included being cured versus all other outcomes including death and the likelihood of dying versus staying alive regardless of the outcome of treatment.

2.4. Outcome and exposures

The first outcome of interest was “cured” defined as 1 if the patient was declared “cured” by the healthcare team or 0 if otherwise. The second outcome was “death” coded 1 if the patient was declared dead and 0 if otherwise. Given the small number of deaths which were 35 in total, we created a nominal outcome, which captured all outcomes and combined them into 3 categories: cured, dead and other outcomes.

2.5. Data collection

Data were extracted from the TB register using structured data collection form from all the TB patients initiated on treatment at this rural public health clinic from 1 January 2013 and 31 December 2015. The data consists of collecting socio-demographic factors (age and gender), TB related factors (categories, pre-treatment smear, type, site, outcome duration, regimen at the baseline) and other factors such as ART initiation and co-trimoxazole prophylaxis, comorbidities, tobacco and alcohol use. A data dictionary was formulated to guide data cleaning and analysis. No personal identifiers were extracted from the manual medical records.

2.6. Data management

Data were entered on excel spread sheet and imported into Stata software. This step was followed by thorough checking to assess missing values. Data were coded into numerical values and continuous data categorised; clean data were imported into Stata version 15 for analysis.

2.7. Data Analysis

The main interest of this study was to compare the likelihood of observing two main outcomes. These included being cured versus all other outcomes including death and the likelihood of dying versus staying alive regardless of the outcome of treatment. Data were summarized and presented in tables. As part of the descriptive statistics for the population under study, summary tables were constructed to describe variables in terms of their frequency distribution. Pearson Chi-square and Fisher Exact tests were used to test association between independent variables and TB outcomes (cured, completed treatment, interrupted treatment, moved, transferred out, failed and died). Given the fact that cured was a fairly common outcome (39%), we fitted a log-binomial regression model to estimate the relative risk of being declared cured versus not being cured (Table 3). Finally, we fitted a multinomial logistic regression model predicting the likelihood of cured and death versus other outcomes. This allowed us to make some predictions on death, as a function of the selected socio-demographic covariates. Odds ratios were computed to assess the association between HIV status and TB mortality. Precision of estimate of the odds ratio was set at 5% significance level. All factors with $p \leq 0.1$ on univariate analysis were further analysed in multiple logistic regression model.

2.8. Ethical considerations

The University of the Witwatersrand Human Research Ethics Committee approved the study. Authority to access medical records was obtained from the Director of Health, Ugu District. No attempt was made to link data to the individual identifier, and data was analysed anonymously.

3. Results

Overall, the study included 508 patients. Of this total, 2 patients were dropped from further analysis because one was transferred to another facility the same day of admission to the hospital and the other one was on prophylaxis TMP-SMX and was HIV negative. Hence, our final analytical sample included 506 patients for whom we had valid data on the TB treatment outcomes. Table 1 described the characteristics of TB patients based on HIV status.

Table 1: Characteristics of TB patients based on HIV status

**Factors**	**HIV Negative n(%)**	**HIV Positive n(%)**	**P-value**
	**178 (37.2)**	**300 (62.8)**	
**Categories of TB**			
New	161 (91.0)	259 (86.3)	0.395¶
RAC	8 (4.5)	25 (8.3)	
RF	5 (2.8)	8 (2.7)	
RI	3 (1.7)	8 (2.7)	
**Pre-treatment smear status**			
Negative	27 (29.7)	41 (30.4)	0.910‡
Positive	64 (70.3)	94 (69.6)	
**Type of Tuberculosis**			
Pulmonary	124 (69.7)	210 (70.0)	0.938‡
Extra-Pulmonary	54 (30.3)	90 (30.0)	
**Site of Tuberculosis**			
Bones/Joints	1 (2.0)	0 (0.0)	0.047*¶
Lymph Nodes	0 (0.0)	4 (4.5)	
Miliary	4 (7.8)	6 (6.7)	
Meningitis	2 (3.9)	17 (19.1)	
Primary	12 (23.5)	17 (19.1)	
Pleura	12 (23.5)	22 (24.7)	
Other Organs	20 (39.2)	23 (25.8)	
**Receiving ART**			
No	172 (100.0)	30 (10.1)	<0.001*¶
Yes	0 (0.0)	267 (89.9)	
**Co-trimoxazole Prophylaxis**			
No	173 (100.0)	22 (7.3)	<0.001*¶

Yes	0 (0.0)	278 (92.7)	
**Outcome duration(days)**	184 (176-203)**	184 (173-209)*	0.7547†
**Comorbidity**			
No	155 (87.1)	286 (95.3)	0.001*
Yes	23 (12.9)	14 (4.7)	
**Regimen type at baseline**			
HRZE	157 (88.7)	280 (93.4)	0.005*¶
HR	15 (8.5)	10 (3.3)	
RHZ	5 (2.8)	3 (1.0)	
HRZES	0 (0.0)	7 (2.3)	

N- Total number in each group; % - row percentages; Numbers may not add-up because of missing variables.

*Significant p-value;

**Median and Inter-quartile ranges (IQR); Test statistic based on ¶Fisher's Exact, ‡Chi-square and Wilcoxson †Rank-sum test.

HIV – Human Immunodeficiency Virus; ART – Antiretroviral therapy; Categories of TB treatment: RAC – retreatment after completion of a previous course without microscopic result; RF – Treatment failure: a patient who, while on treatment, remained or became again smear positive five months or later after commencing treatment; RI – Treatment after interruption: a patient whose treatment is interrupted for two or more months and who returned to the health service.

HRZE – Isoniazid, Rifampicin, Pyrazinamide and Ethambutol for New adult patients; HRZ – Isoniazid, Rifampicin and Pyrazinamide for New Paediatric patients; HRZES – Isoniazid, Rifampicin, Pyrazinamide, Ethambutol and Streptomycin for Retreatment patients.

Table 2 revealed that 39.13% (n=198) were reported as being cured at the end of treatment. The remaining 60.87% patients were classified as dead or alive, but not cured. Some patients were transferred to other facilities, whereas other were classified as treatment interrupted, transferred to another district, treatment outcome unknown, treatment completed and moved to another facility in the same district. Table 2 also showed that age, tobacco, and alcohol uses were significantly associated with TB cured. For instance, at younger age patients were less likely to be cured (22.62% of those less than 20 years of age) compared to older patients. Among tobacco users, we found that about 60% were TB cured, whereas among non-tobacco users only 37% were cured. Likewise, the findings estimated that alcohol users, had higher

proportion of being TB cured (57.14% of all alcohol users) than non-alcohol users' patients (38.08%).

The overall TB related mortality rate was 6.92% (n=35 patients). As expected, the mortality rate was significantly associated with HIV status. For instance, the mortality rate was higher among HIV positive patients (9.67%, i.e. 29 patients out of 300 patients) compared to HIV negative patients among whom only for 2.91% was reported (6 patients out of 206 HIV negative patients). Given the small sample size for the mortality rate, this was impractical to perform further analysis and obtain reliable estimates.

Table 2. Characteristics of TB patients based on TB cured outcomes

Factors	Not Cured n (%)	Cured n (%)	P-value	Alive (cured, transferred, other) n (%)	Dead n (%)	p-value
	**308 (60.87)**	**198 (39.13)**		**471 (93.08)**	**35 (6.92)**	
**Duration on treatment[1]**	5.55 (0.16)	6.15 (0.13)	0.009*	6.01 (0.11)	2.74 (0.44)	0.001*
**HIV Status**						
Negative	123 (59.71)	83 (40.29)	0.658	200 (97.09)	6 (2.91)	0.004*
Positive	185 (61.67)	115 (38.33)		271 (90.33)	29 (9.67)	
**Age-group**						
<20	65 (77.38)	19 (22.62)	0.018*	82 (97.62)	2 (2.38)	0.101[F]
20 – 29	78 (58.21)	56 (41.79)		124 (92.54)	10 (7.46)	
30 – 39	72 (55.38)	58 (44.62)		120 (92.31)	10 (7.69)	
40 – 49	46 (58.97)	32 (41.03)		75 (96.15)	3 (3.85)	
50+	47 (58.75)	33 (41.25)		70 (87.50)	10 (12.50)	
**Sex**						
Female	138 (61.61)	86 (38.39)	0.762	205 (91.52)	19 (8.48)	0.216
Male	170 (60.28)	112 (39.72)		266 (94.33)	16 (5.67)	
**Comorbidity**						

No	286 (60.98)	183 (39.02)	0.855	438 (93.39)	31 (6.61)	0.311[F]
Yes	22 (59.46)	15 (40.54)		33 (89.19)	4 (10.81)	
**Tobacco Use**						
No	292 (62.66)	174 (37.34)	0.005*	432 (92.70)	34 (7.30)	0.345[F]
Yes	16 (40.00)	24 (60.00)		39 (97.50)	1 (2.50)	
**Alcohol Use**						
No	296 (61.92)	182 (38.08)	0.045*	443 (92.68)	35 (7.32)	0.246[F]
Yes	12 (42.86)	16 (57.14)		28 (100.00)	0 (0.00)	
**Substance Use**						
No	292 (60.08)	194 (39.92)	0.101	452 (93.00)	34 (7.00)	1.00[F]
Yes	16 (80.00)	4 (20.00)		19 (95.00)	1 (5.00)	

Note: [1] Duration of treatment in months
All p-value from Pearson Chi-square test, unless otherwise determined.
** p-value < 0.05; [F]indicates results from Fisher Exact test*

Table 3 below presented the results from the bivariate and multivariable log-binomial model, which sought to identify covariates associated with cure event. These included duration of treatment, HIV status, age, comorbidity, tobacco, and alcohol use. All these covariates were shown to be associated with being cured, except HIV status. However, since HIV status was our main exposure of interest, we kept it in the models.

We also found that, HIV positive status did not affect the likelihood of TB cured after treatment. Results from the analysis suggested that HIV positive patients were less likely to be cured from TB compared to HIV negative patients. However, we found no evidence that this result was statistically significant (RR: 0.88; 95% C.I.: 0.69 – 1.11). The course of TB treatment was associated with TB cured. For instance, an additional month on TB treatment was associated with 3% increased risk of TB cured (RR: 1.03; 95% C.I.: 1.01 – 1.06).

In the bivariate models revealed that tobacco (RR: 1.60(1.22 – 2.12)) and alcohol users (RR: 1.5(1.07 – 2.11)) had a higher likelihood of being cured. However, these

results did not hold true in the covariate adjusted model. We compared the full spectrum of all possible outcomes experienced by the patients.

Table 3. Bivariate and covariate log-binomial regression model predicting relative risk of being cured as a function of sociodemographic covariates

Factors	Bivariate model	Covariate adjusted model
	**RR (95% C.I.)**	**RR (95% C.I.)**
**Duration on treatment**	**1.03(1.01 – 1.06)**	**1.03(1.01 – 1.06)**
**HIV Status**		
Negative	Ref	Ref
Positive	0.95 (0.76-1.19)	0.88(0.69-1.11)
**Age-group**		
<20	Ref	Ref
20 – 29	1.85(1.19 – 2.88)	1.82(1.15 – 2.91)
30 – 39	1.97(1.27 -3.06)	2.00(1.26 – 3.17)
40 – 49	1.81(1.13 – 2.92)	1.79(1.10 – 2.91)
50+	1.82(1.13 – 2.93)	1.77(1.09 – 2.85)
**Sex**		
Female	Ref	Ref
Male	1.03(0.83 – 1.29)	-
**Comorbidity**		
No	Ref	-
Yes	1.04(0.69 – 1.56)	-
**Tobacco Use**		
No	Ref	Ref
Yes	1.60(1.22 – 2.12)	1.54(0.95 – 2.48)
**Alcohol Use**		
No	Ref	Ref
Yes	1.5(1.07 – 2.11)	0.93(0.51 – 1.69)

Table 4 below present's results from the multinomial logistic regression models with three levels of outcomes: cured, dead and other. Results from the multinomial logistic regression models suggest that HIV status and age were associated with both death and cured. In addition, duration of treatment was associated with death, while tobacco use was associated with cured. We found that longer treatment period was associated

with lower risk of death (RRR: 0.56; 95% C.I.: 0.47 – 0.66) compared to other outcomes, whereas HIV positive patients had higher probability of death (RRR: 3.73; 95% C.I.: 1.24 – 11.19) compared to other outcomes.

We also found that younger age had lower likelihood of being cured compared to older ages. For instance, compared to younger age (i.e. less than 20 years old), patients with 50+ years of age had about 3-fold increased likelihood of being cured (RRR: 3.01; 95% C.I. 1.44 – 6.27) compared to reporting other outcomes.

Table 4. Bivariate and covariate adjusted multinomial logistic regression model predicting cure and death as a function of sociodemographic covariates

**Factors**	**Bivariate multinomial**		**Covariate adjusted multinomial**	
	**Cured versus Other outcomes***	**Died versus Other outcomes**	**Cured versus other outcomes**	**Died versus other outcomes**
	RRR (95% C.I.)	RRR (95% C.I.)	RRR (95% C.I.)	RRR (95% C.I.)
**Duration on treatment**	1.04 (0.97 – 1.13)	0.54 (0.46 – 1.13)	1.05 (0.97 – 1.13)	0.56 (0.47 – 0.66)
**HIV Status**				
Negative	Ref	Ref	Ref	Ref
Positive	1.04 (0.72 – 1.50)	3.63 (1.46 – 9.02)	0.80 (0.52 – 1.22)	3.73 (1.24 – 11.19)
**Age group**				
<20	Ref	Ref	Ref	Ref
20 – 29	2.73 (1.46 – 5.09)	4.63 (0.98 – 21.97)	2.76 (1.44 – 5.28)	2.40 (0.45 – 12.78)
30 – 39	3.10 (1.66 – 5.80)	5.08 (1.07 – 24.13)	3.34 (1.69 – 6.62)	2.18 (0.39 – 12.29)
40 – 49	2.47 (1.24 – 4.91)	2.20 (0.35 – 13.71)	2.63 (1.25 – 5.54)	1.46 (0.20 – 10.83)
50+	2.96 (1.48 – 5.93)	8.51 (1.77 – 40.98)	3.01 (1.44 – 6.27)	4.99 (0.88 – 28.50)
**Sex**				
Female	Ref	Ref	Ref	Ref
Male	1.01 (0.70 – 1.46)	0.65 (0.32 – 1.32)	0.81 (0.54 – 1.20)	0.61 (0.26 – 1.46)
**Co-morbidity**				
No	Ref	Ref	Ref	Ref
Yes	1.16 (0.57 – 2.36)	1.83 (0.58 – 5.75)	0.90 (0.41 – 1.96)	1.30 (0.29 – 5.80)
**Tobacco Use**				
No	Ref	Ref	Ref	Ref
Yes	2.37 (1.21 – 4.65)	0.51 (0.06 – 3.95)	2.24 (1.11 – 4.52)	0.56 (0.06 – 4.96)

**other outcomes include…. Alive, transferred, defaulted etc*

4. Discussion

This study evaluated the effect of HIV status and antiretroviral treatment on the treatment outcome of Tuberculosis patients. The study comprised of 282 males (55.7) and 224 females (44.3) with a median age of 32 (IQR: 23-43) years. The main outcomes of the study were categorized as cured or dead. This study found that 39.13% (198/506) of the patients were cured of Tuberculosis, of which 115 were HIV positive. The overall TB related mortality rate was 6.92% (n=35 patients). Our study also revealed that the mortality rate was significantly associated with HIV status. For instance, the mortality rate was higher among HIV positive patients (9.67%, i.e. 29 patients out of 300 patients) compared to HIV negative patients among whom only for 2.91% was reported (6 patients out of 206 HIV negative patients).

In reviewing other studies conducted in rural Africa, HIV status did not appear to have any effect on the treatment of tuberculosis and this could be due to a lack of data for some of the participants in a study conducted in Ghana [27]. However, the same study found that HIV status was associated with death [27]. HIV-positive patients were 3.73 times more likely to die than HIV-negative patients (RRR: 3.73, 95% CI: 1.24-11.19) [27]. This is expected, as other studies have also shown a higher mortality rate among HIV/TB co-infected individuals [27]. Another cohort in Tanzania found an EPTB prevalence of 5.6 per cent with a high mortality rate of 14.3 per cent (25/175) and a combined death/LTFU rate of 46.8 per cent among people living with HIV [28]. Patients with EPTB not receiving ART and >45 years of age were at higher risk for poor outcomes [28]. There were no differences in treatment success rates for HIV-positive TB patients in ART and HIV-negative pre-and post-integration patients in Kenya [29].

Age was reported as significantly associated with being cured. Younger patients of less than 20 years were less likely to be cured compared to older patients 20 years

and above [30]. These results differ from those of a study conducted in Zimbabwe where elderly patients had a higher risk of not being cured, the most common outcome was death [30]. The same study revealed that older people understood the importance of taking their medication for better health and thus they were more likely to achieve positive outcomes as they take their treatment regularly [30]. Other age groups were already familiar with treatments from co-morbidities such as diabetes hence they did not find it difficult to adhere to their Tuberculosis medication [30].

A meta-analysis of high-quality studies showed that smoking tobacco was noted to have an effect on the outcome of Tuberculosis treatment as smokers had a higher chance of being cured compared with non-smokers (OR of 2.6 (95%CI 2.1–3.4) [31]. Further, the associations between smoking and TB mortality showed a pooled OR of 1.3 (95%CI 1.1–1.6) [31]. Another large systematic review showed that cigarette smoking was significantly linked with poor tuberculosis treatment outcomes [32]. The findings of our study confirmed previous studies undertaken on the association between smoking tobacco and TB cured.

Finally, a large cohort in Western Cape showed that concomitant diseases were not statistically significant with treatment outcomes [33]. This was well-founded in our study. However, the adjusted risk ratio indicated that treatment categories and patients with comorbidity disorders such as diabetes, liver disease, renal failure, hepatitis and silicosis were all significantly associated with unsuccessful TB treatment [33].

5. Conclusion

The main factors associated with TB outcomes were duration of treatment, age, smoking, alcohol use, and HIV status. The duration of treatment was associated with the cure, transfer and death of TB. HIV positive status, Tobacco and alcohol use have appeared to decrease the TB cure. On the other hand, the age has shown an important effect on the treatment of TB. In addition, there was no evidence of association between sex, comorbidity, substance use, and TB cured and died. In sum, this study revealed that the cured rate was below district and provincial targets at this facility. Despite a high ART coverage, HIV status was a strong predictor of the unfavourable TB treatment outcomes.

6. Supporting information

S1 Dataset (XLSX)

7. **Acknowledgements:** The authors would like to thanks the staff of Elim Clinic for the support provided during the duration of this study.

8. Author Contributions

Conceptualization: Peter S Nyasulu, Jabulani Ncayiyana

Data curation: Peter S Nyasulu, Lovemore N Sigwadhi, Emery Ngasama, Teye Umanah, Jabulani Ncayiyana

Formal analysis: Lovemore N Sigwadhi, Emery Ngasama, Lovelyn U Ozougwu, Ruvimbo BC Nhandara, Teye Umanah, Birhanu T Ayele

Investigation: Peter S Nyasulu, Emery Ngasama, Jacques L Tamuzi, Birhanu T Ayele, Jabulani Ncayiyana.

Methodology: Peter S Nyasulu, Emery Ngasama, Jacques L Tamuzi, Lovemore N Sigwadi, Birhanu T Ayele, Jabulani Ncayiyana.

Supervision: Peter S Nyasulu, Birhanu T Ayele, Jabulani Ncayiyana

Writing – original draft: Peter S Nyasulu, Jacques L Tamuzi, Lovelyn U Ozougwu Ruvimbo BC Nhandara

Writing – review & editing: Peter S Nyasulu, Emery Ngasama, Jacques L Tamuzi, Lovemore N Sigwadhi, Lovelyn U Ozougwu, Ruvimbo BC Nhandara, Birhanu T Ayele, Teye Umanah, Jabulani Ncayiyana.

9. References

1. World Health Organization. Global tuberculosis report, 2020. Available from: https://apps.who.int/iris/bitstream/handle/10665/336069/9789240013131-eng.pdf.
2. Department of Health (Republic of South Africa). National Tuberculosis Management Guidelines, 2009. Available from: https://www.tbonline.info/media/uploads/documents/south_african_national_tuberculosis_management_guidelines_%282009%29.pdf
3. World Health Organization. Global tuberculosis report (executive summary), 2018. Available from: https://www.who.int/tb/publications/global_report/tb18_ExecSum_web_4Oct18.pdf?ua=1#:~:text=Globally%2C%20the%20absolute%20number%20of,of%20the%20End%20TB%20Strategy).
4. Roy E, Lowrie DB, Jolles SR. Current strategies in TB immunotherapy. Current Molecular Medicine 2007; 7(4):373-86.

5. Kalonji D, Mahomed OH. Health system challenges affecting HIV and tuberculosis integration at primary healthcare clinics in Durban, South Africa. African journal of primary health care & family medicine 2019;11(1):1-7.
6. Manosuthi W, Wiboonchutikul S, Sungkanuparph S. Integrated therapy for HIV and tuberculosis. AIDS Res Ther 2016; 13(1):1-12.
7. Naidoo K, Gengiah S, Yende-Zuma N, Padayatchi N, Barker P, Nunn A, et al. Addressing challenges in scaling up TB and HIV treatment integration in rural primary healthcare clinics in South Africa (SUTHI): a cluster randomized controlled trial protocol. Implementation Science 2017; 12(1):129.
8. Gupta Rishi K, Lucas Sebastian B, Fielding Katherine L, Lawn Stephen D. Prevalence of tuberculosis in post-mortem studies of HIV-infected adults and children in resource-limited settings: a systematic review and meta-analysis. AIDS (London, England) 2015; 29(15):1987-2002.
9. Tamuzi JL, Ayele BT, Shumba CS, Adetokunboh OO, Uwimana-Nicol J, Haile ZT, et al. Implications of COVID-19 in high burden countries for HIV/TB: A systematic review of evidence. BMC infectious diseases 2020; 20(1):1-18.
10. World Health Organization. HIV-Associated Tuberculosis, 2019. Available from: https://www.who.int/tb/areas-of-work/tb-hiv/tbhiv_factsheet.pdf?ua=1.
11. KwaZulu-Natal Department of Health. KwaZulu-Natal Department of Health-Strategic Plan 2014/15 - 2018/19. Pietermaritzburg: KwaZulu-Natal Department of Health; 2015. Available from: http://www.kznhealth.gov.za/Annual-Performance-Plan-2015-16.pdf
12. Pillay-van WV, Bradshaw D. Mortality and socioeconomic status: The vicious cycle between poverty and ill health. Lancet Glob Health 2017; 5(9): e851–e852.

13. Statistic South Africa. Mid-year population estimates, 2019. Available from: https://www.statssa.gov.za/publications/P0302/P03022019.pdf.

14. Nanoo A, Izu A, Ismail NA, Ihekweazu C, Abubakar I, Mametja D, et al. Nationwide and regional incidence of microbiologically confirmed pulmonary tuberculosis in South Africa, 2004-12: a time series analysis. The Lancet infectious diseases 2015; 15(9):1066-76.

15. Sloot R, Maarman GJ, Osman M, Marx FM. Variation in HIV prevalence and the population-level effects of antiretroviral therapy in reducing tuberculosis incidence in South Africa. South African Medical Journal 2018;108(8):606.

16. Tomita A, Smith CM, Lessells RJ, Pym A, Grant AD, de Oliveira T, et al. Space-time clustering of recently-diagnosed tuberculosis and impact of ART scale-up: evidence from an HIV hyper-endemic rural South African population. Scientific reports 2019;9(1):1-9.

17. Health System Trust. Durban. District Health Barometer 2015/16. https://www.hst.org.za/publications/District%20Health%20Barometers/District%20Health%20Barometer%202015_16.pdf

18. Khan PY, Crampin AC, Mzembe T, Koole O, Fielding K L, Kranzer K, et al. Does antiretroviral treatment increase the infectiousness of smear-positive pulmonary tuberculosis? The international journal of tuberculosis and lung disease. The official journal of the International Union against Tuberculosis and Lung Disease 2017;21(11):1147-54.

19. van Halsema CL, Fielding KL, Chihota VN, George EC, Lewis JJ, Churchyard GJ, et al. Brief report: the effect of antiretroviral therapy and CD4 count on markers of infectiousness in HIV-associated tuberculosis. JAIDS Journal of Acquired Immune Deficiency Syndromes 2015;70(1):104-8.

20. Abdool KSS, Naidoo K, Grobler A, Padayatchi N, Baxter C, Gray AL, et al. Integration of antiretroviral therapy with tuberculosis treatment. New England Journal of Medicine 2011;365(16):1492-501.

21. Blanc FX, Sok T, Laureillard D, Borand L, Rekacewicz C, Nerrienet E, et al. Earlier versus later start of antiretroviral therapy in HIV-infected adults with tuberculosis. New England Journal of Medicine 2011; 365(16):1471-81.

22. Havlir DV, Kendall MA, Ive P, Kumwenda J, Swindells S, Qasba SS, et al. Timing of antiretroviral therapy for HIV-1 infection and tuberculosis. New England Journal of Medicine 2011; 365(16):1482-91.

23. World Health Organization. WHO Global Tuberculosis Report - South Africa profile, 2019. Available from: file:///D:/Files/The%20burden%20of%20TB-COVID-19%20in%20Sub-Saharan%20Africa/2019%20WHO%20Global%20Tuberculosis%20Report%20-%20South%20Africa%20profile.pdf

24. Scott V, Sanders D. Evaluation of how integrated HIV and TB programs are implemented in South Africa and the implications for rural-urban equity 2013. Available from: http://repository.uwc.ac.za/handle/10566/1111

25. Kwan CK, Ernst JD. HIV and tuberculosis: a deadly human syndemic. Clin MicrobiolRev 2011; 24(2):351-76.

26. Houlihan CF, Mutevedzi PC, Lessells RJ et al. The tuberculosis challenge in a rural South African HIV programme. BMC infectious diseases 2010; 10(1): 23.

27. Ogyiri L, Lartey M, Ojewale O, Adjei AA, Kwara A, Adanu RM, et al. Effect of HIV infection on TB treatment outcomes and time to mortality in two urban hospitals in Ghana-a retrospective cohort study. The Pan African Medical Journal 2019; 32: 206.

28. Arpagaus A, Franzeck FC, Sikalengo G, Ndege R, Mnzava D, Rohacek M, et al. Extrapulmonary tuberculosis in HIV-infected patients in rural Tanzania: The prospective Kilombero and Ulanga antiretroviral cohort. Plos one 2020;15(3): e0229875.
29. Owiti P, Zachariah R, Bissell K, Kumar A M V, Diero L, Carter E J, et al. Integrating tuberculosis and HIV services in rural Kenya: uptake and outcomes. Public health action 2015; 5(1):36-44.
30. Ncube RT, Takarinda KC, Zishiri C, Van den Boogaard W, Mlilo N, Chiteve C, et al. Age-stratified tuberculosis treatment outcomes in Zimbabwe: are we paying attention to the most vulnerable? Public health action 2017;7(3):212-7.
31. Slama K, Chiang CY, Enarson DA, Hassmiller K, Fanning A, Gupta P and Ray C. Tobacco and tuberculosis: a qualitative systematic review and meta-analysis. The International Journal of Tuberculosis and Lung Disease 2007, 11(10): 1049-1061.
32. Burusie A, Enquesilassie F, Addissie A, Dessalegn B, Lamaro T. Effect of smoking on tuberculosis treatment outcomes: A systematic review and meta-analysis. PloS one 2020;15(9): e0239333.
33. Azeez A, Ndege J and Mutambayi R. Associated factors with unsuccessful tuberculosis treatment outcomes among tuberculosis/HIV coinfected patients with drug-resistant tuberculosis. International Journal of Mycobacteriology 2018; 7(4): 347.

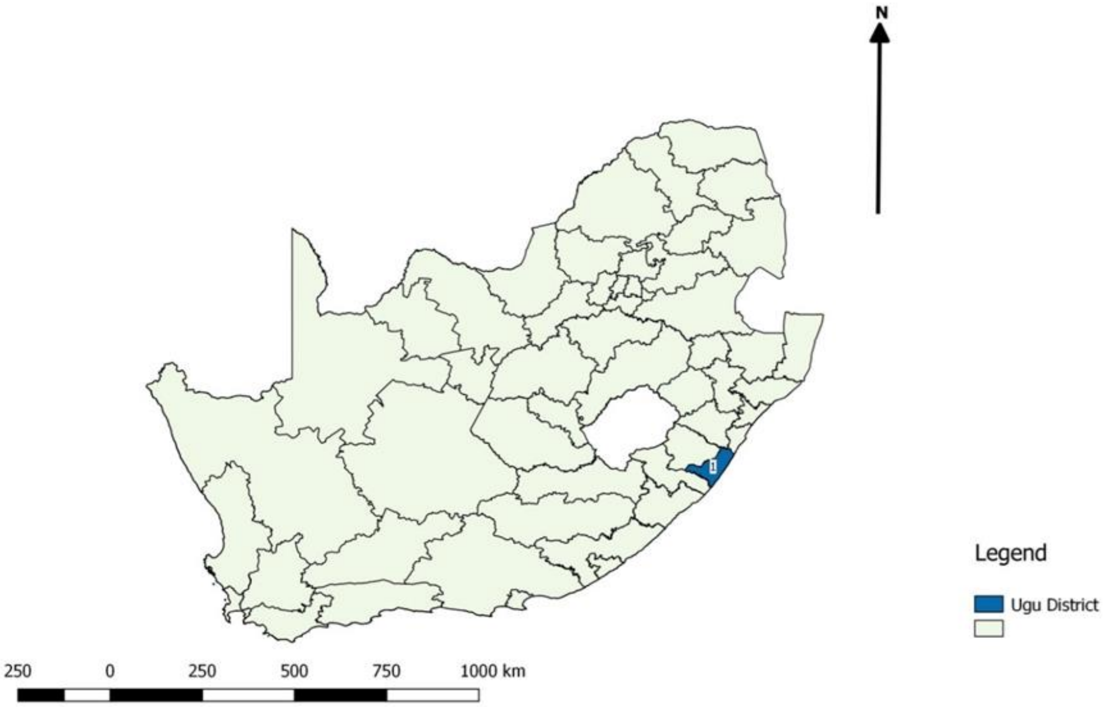

Figure 1: Map of study setting: Ugu disctrict in KwaZulu Natal/South Africa
