# Supplementary material for: Effect of HIV status and antiretroviral treatment on treatment outcomes of tuberculosis patients in a rural primary healthcare clinic in South Africa
Source: PLoS One. 2022 Oct 12;17(10):e0274549. doi: 10.1371/journal.pone.0274549 (PMC9555649; doi:10.1371/journal.pone.0274549)
Supplement: Attachment — Submitted filename: PONE-D-21-05746_R1.pdf [file pone.0274549.s004.pdf]

PLOS ONE

Effect of HIV status and antiretroviral treatment on treatment outcomes of tuberculosis patients in a rural primary healthcare clinic in South Africa

--Manuscript Draft--

**Manuscript Number:**	PONE-D-21-05746R1
**Article Type:**	Research Article
**Full Title:**	Effect of HIV status and antiretroviral treatment on treatment outcomes of tuberculosis patients in a rural primary healthcare clinic in South Africa
**Short Title:**	HIV Infection and treatment outcomes among Tuberculosis patients in rural South Africa
**Corresponding Author:**	Peter S Nyasulu, PhD<br>Stellenbosch University Faculty of Medicine and Health Sciences<br>Cape Town, Western Cape SOUTH AFRICA
**Keywords:**	TB; Treatment outcomes; Co-infection; HIV Status, KwaZulu Natal province, South Africa
**Abstract:**	Background<br>Tuberculosis (TB) remains the leading cause of death among human immunodeficiency virus (HIV) infected individuals in South Africa. Despite the implementation of HIV/TB integration services at primary healthcare facility level, the effect of HIV on TB treatment outcomes has not been well investigated. To provide evidence base for TB treatment outcome improvement to meet End TB Strategy goal, we assessed the effect of HIV status on treatment outcomes of TB patients at a rural clinic in the Ugu Health District, South Africa.<br>Methods<br>We reviewed medical records involving a cohort of 508 TB patients registered for treatment between 1 January 2013 and 31 December 2015 at rural public sector clinic in KwaZulu-Natal province, South Africa. Data were extracted from National TB Programme clinic cards and the TB case registers routinely maintained at study sites. The effect of HIV status on TB treatment outcomes was determined by using multinomial logistic regression. Estimates used were relative risk ratio (RRR) at 95% confidence intervals (95%CI).<br>Results<br>A total of 506 patients were included in the analysis. Majority of the patients (88%) were new TB cases, 70% had pulmonary TB and 59% were co-infected with HIV. Most of HIV positive patients were on antiretroviral therapy (ART) (90% (n=268)). About 82% had successful treatment outcome (cured 39.1% (n=198) and completed treatment (42.9% (n=217)), 7% (n=39) died 0.6% (n=3) failed treatment, 3.9% (n=20) defaulted treatment and the rest (6.6% (n=33)) were transferred out of the facility. Using completed treatment as reference, HIV positive patients not on ART relative to negative patients were more likely to have unsuccessful outcomes [RRR, 5.41; 95%CI, 2.11–13.86].<br>Conclusions<br>When compared HIV positive and HIV negative status, antiretroviral treatment had no effect on the likelihood of TB cure in rural primary care. The TB mortality rate in HIV positive patients, on the other hand, was higher than in HIV negative patients. Various HIV/TB indicators should be reviewed, and gaps filled to achieve the “End TB strategy” in rural South Africa.
**Order of Authors:**	Peter S Nyasulu, PhD
	Emery L Ngasama
	Jacques Lukenze Tamuzi
	Lovemore N Sigwadi
	Lovelyn U Ozougwu
	Ruvimbo BC Nhandara

Powered by Editorial Manager® and ProduXion Manager® from Aries Systems Corporation

	Birhanu T Ayele
	Teye Umanah
	Jabulani Ncayiyana
**Response to Reviewers:**	Reviewers' responses to reviewers<br><br>Title: Effect of HIV status and antiretroviral treatment on treatment outcomes of tuberculosis patients in a rural primary healthcare clinic in South Africa<br><br><br>Journal Requirements:<br>Comment 1. Please ensure that your manuscript meets PLOS ONE's style requirements, including those for file naming. The PLOS ONE style templates can be found at<br>https://journals.plos.org/plosone/s/file?id=wjVg/PLOSOne_formatting_sample_main_body.pdf and<br>https://journals.plos.org/plosone/s/file?id=ba62/PLOSOne_formatting_sample_title_authors_affiliations.pdf<br>Response: Thanks, we have revised the manuscript in accordance with the PLOS One formatting sample.<br>Comment 2. In the ethics statement in the manuscript and in the online submission form, please provide additional information about the patient records used in your retrospective study, including a) whether all data were fully anonymized before you accessed them; b) the date range (month and year) during which patients' medical records were accessed; c) the date range (month and year) during which patients whose medical records were selected for this study sought treatment. If the ethics committee waived the need for informed consent, or patients provided informed written consent to have data from their medical records used in research, please include this information.<br>Response: Thanks for the comments and suggestions. Data collection for this study commenced from the 1st of September 2016 and ended on 31st of October 2016. The data clerk was contracted for a 2-month period to enter as well as do quality control of the data entered. Data were abstracted directly from registers of TB patients who were initiated on TB treatment from 1 January 2013 to 31 December 2015. Furthermore, TB patients registered in the TB register in 2012 and died in 2013 were also included in the data extraction. (Line 147 to 152 Page 8). The data entry clerk was under strict instruction to enter data anonymously from the TB registers onto the database. No identifiers were extracted from the TB registers. It should be noted that this data were extracted in 2016 of patients who received service for TB treatment almost 3 years previously. As a result it was not possible to get patient consent as these were historical records and patients had been out of the system for 3 years already by the time the study was being conducted, so we had to seek waiver of consent to use the data as we believed that we could generate informative data that would shape policy and treatment guidelines in the management of TB at a time when expanding access to TB/HIV collaborative treatment was actively being rolled out by the department of health as a successful model of clinical care to minimise poor outcomes. Ethical approval was granted by the Monash University Human Research Ethics Committee (approval number: CF16/2803- 2014001548). Permission to conduct the study at Elim Primary Health Care Clinic was obtained from the Director of in Ugu Health District, KwaZulu Natal.<br><br>Comment 3. Thank you for stating the following financial disclosure:<br>Response: N/A<br>At this time, please address the following queries:<br>a) Please clarify the sources of funding (financial or material support) for your study. List the grants or organizations that supported your study, including funding received from your institution. N/A<br>b) State what role the funders took in the study. If the funders had no role in your study, please state: "The funders had no role in study design, data collection and analysis, decision to publish, or preparation of the manuscript." N/A<br>c) If any authors received a salary from any of your funders, please state which authors and which funders. N/A<br>d) If you did not receive any funding for this study, please state: "The authors received

no specific funding for this work."
Please include your amended statements within your cover letter; we will change the online submission form on your behalf.
Response: In track change document on page 26, Line 492. Thanks, we state this: "Financial Disclosure: The author(s) received no specific funding for this work".

Comment 4. In your Data Availability statement, you have not specified where the minimal data set underlying the results described in your manuscript can be found. PLOS defines a study's minimal data set as the underlying data used to reach the conclusions drawn in the manuscript and any additional data required to replicate the reported study findings in their entirety. All PLOS journals require that the minimal data set be made fully available. For more information about our data policy, please see http://journals.plos.org/plosone/s/data-availability.
Upon re-submitting your revised manuscript, please upload your study's minimal underlying data set as either Supporting Information files or to a stable, public repository and include the relevant URLs, DOIs, or accession numbers within your revised cover letter. For a list of acceptable repositories, please see http://journals.plos.org/plosone/s/data-availability#loc-recommended-repositories. Any potentially identifying patient information must be fully anonymized.
Important: If there are ethical or legal restrictions to sharing your data publicly, please explain these restrictions in detail. Please see our guidelines for more information on what we consider unacceptable restrictions to publicly sharing data: http://journals.plos.org/plosone/s/data-availability#loc-unacceptable-data-access-restrictions. Note that it is not acceptable for the authors to be the sole named individuals responsible for ensuring data access.
We will update your Data Availability statement to reflect the information you provide in your cover letter.
Response: In track change document on page 23, lines 423-424. Thanks, Dataset is available upon request by submitting the request form to the corresponding author (email: pnyasulu@sun.ac.za).

Comment 5. We note that Figure 1 in your submission contain map images which may be copyrighted. All PLOS content is published under the Creative Commons Attribution License (CC BY 4.0), which means that the manuscript, images, and Supporting Information files will be freely available online, and any third party is permitted to access, download, copy, distribute, and use these materials in any way, even commercially, with proper attribution. For these reasons, we cannot publish previously copyrighted maps or satellite images created using proprietary data, such as Google software (Google Maps, Street View, and Earth). For more information, see our copyright guidelines: http://journals.plos.org/plosone/s/licenses-and-copyright.

We require you to either (1) present written permission from the copyright holder to publish these figures specifically under the CC BY 4.0 license, or (2) remove the figures from your submission:

a. You may seek permission from the original copyright holder of Figure 1 to publish the content specifically under the CC BY 4.0 license.

We recommend that you contact the original copyright holder with the Content Permission Form (http://journals.plos.org/plosone/s/file?id=7c09/content-permission-form.pdf) and the following text:
"I request permission for the open-access journal PLOS ONE to publish XXX under the Creative Commons Attribution License (CCAL) CC BY 4.0 (http://creativecommons.org/licenses/by/4.0/). Please be aware that this license allows unrestricted use and distribution, even commercially, by third parties. Please reply and provide explicit written permission to publish XXX under a CC BY license and complete the attached form."

Please upload the completed Content Permission Form or other proof of granted permissions as an "Other" file with your submission.

In the figure caption of the copyrighted figure, please include the following text: "Reprinted from [ref] under a CC BY license, with permission from [name of publisher], original copyright [original copyright year]."

Powered by Editorial Manager® and ProduXion Manager® from Aries Systems Corporation

b. If you are unable to obtain permission from the original copyright holder to publish these figures under the CC BY 4.0 license or if the copyright holder's requirements are incompatible with the CC BY 4.0 license, please either i) remove the figure or ii) supply a replacement figure that complies with the CC BY 4.0 license. Please check copyright information on all replacement figures and update the figure caption with source information. If applicable, please specify in the figure caption text when a figure is similar but not identical to the original image and is therefore for illustrative purposes only.
The following resources for replacing copyrighted map figures may be helpful:

USGS National Map Viewer (public domain): http://viewer.nationalmap.gov/viewer/
The Gateway to Astronaut Photography of Earth (public domain): http://eol.jsc.nasa.gov/sseop/clickmap/
Maps at the CIA (public domain): https://www.cia.gov/library/publications/the-world-factbook/index.html and https://www.cia.gov/library/publications/cia-maps-publications/index.html
NASA Earth Observatory (public domain): http://earthobservatory.nasa.gov/
Landsat: http://landsat.visibleearth.nasa.gov/
USGS EROS (Earth Resources Observatory and Science (EROS) Centre) (public domain): http://eros.usgs.gov/#
Natural Earth (public domain): http://www.naturalearthdata.com/

Response: Thanks for this comment. The map was created using a shapefile from the Africa map library software, which was then imported into the Quantum Geographic information system (QGIS) software for georeferencing of the Ugu district. Figure 1 is licensed under a CC BY 4.0

Comment 6. Please upload a copy of Supporting Information S1 Dataset which you refer to in your text on page 18.

Response: In track change document on page 21 and lines 399. Thanks, we have uploaded the raw data for the study.

Reviewer #1
Comment 1: although this statement maybe correct, it cannot be a conclusion at the level of the abstract as the reader has no comparator.
Response: In track change document on page 3 and lines 46 to 49. The conclusion is now: "when compared HIV positive and HIV negative status, antiretroviral treatment had no effect on the likelihood of TB cure in rural primary care. The TB mortality rate in HIV positive patients, on the other hand, was higher than in HIV negative patients".

Comment 2: based on this what is your recommendation.
Response: In track change document on page 3 and lines 49 to 50. We recommend this: "Various HIV/TB indicators should be reviewed, and gaps filled in order to achieve the "End TB strategy" in rural South Africa".

Comment 3: comma
Response: In track change document on page 4 and line 64, the comma has been inserted.

Comment 4: I suggest that paragraph 2 is integrated at this point to improve the flow and readability of the manuscript.
Response: In track change document on page 4 and lines 65-71. Thanks, paragraph 2 has been integrated as suggested.

Comment 5: I think that this sentence is redundant information.
Response: In track change document on page 4 and lines 75-77. Thanks, this sentence has been written more clearly in the background.

Comment 6: There are two concepts being discussed in this sentence- decrease in mortality and increased incidence. however, the sentence needs multiple readings to make sense. therefore, I suggest that the sentence is separated.
Response: In track change document on page 4 and lines 63-66. We have separated

the two concepts. It now reads: “The increase in incidence is also attributed to the development of multidrug resistant (MDR) and extremely drug resistant (XDR) strains of Mycobacterium tuberculosis [34]. Both MDR/XDR-TB are the causes of high TB mortality [4]”.

Comment 7: This sentence appears out of sync as the previous discussion only relate to mortality and this sentence intends on providing reason for increased risk for TB.
Response: The sentence has been deleted and removed.

Comment 8: Which countries as the authors quoting facility based postmortem findings.
Response: In track change document on pages 4-5 and lines 75-77. Thanks, we have listed the countries reporting post-mortem TB rate. It now reads: “such as South Africa [9-11], Botswana [11, 12], Zimbabwe [11, 13, 14], Mozambique [11, 15], Uganda [11, 16] and Kenya [11, 17]”.

Comment 9: I am sure that the WHO global report on TB provides data in resource limited countries.
Response: In track change document on page 5 and lines 77. Thanks, we have provided TB data in Africa. “In contrast, the WHO reported 16% of HIV/TB related death in Africa [1]”.
Comment 10: please update this data as later information is available.
Response: In track change document on pages 5 and lines 80-81. Thanks, this data has been updated. “TB accounted for the third highest number of deaths in 2018 (6 %; n = 454 014)”
Comment 11: specific data for Ugu district
Response: In track change document on page 5 and lines 87-88. “Ugu district reported 60.5% of TB/HIV co-infection rate [25]”.
Comment 12: provide information on South Africa antiretroviral treatment programme in terms of number of patients on treatment and accessibility to treatment.
Response: In track change document on pages 6 and lines 89-90. Thanks, we have reported this: “According to recent data, 90% of people are aware of their HIV status of these 68% are on antiretroviral therapy (ART) of which 87% are virally suppressed in SA [26]”.
Comment 13: this paragraph is out of sync in building the case. it may be more suited to a discussion
Response: In track change document on pages 19-20, lines 360-367. Thanks, this paragraph has been moved to the discussion section.

Comment 14: with a view to?
Response: In track change document on page 7 and lines 141. Thanks, we state as follows: “with a view to Ugu district”.

Comment 15: the author needs to separate study design, population and study setting
Response: In track change document on pages 8-9 and lines 122-142.Thanks, we have separated the study design, population and study setting. It is now reads:
“Study design
This retrospective cohort study of TB patients initiated on TB treatment was conducted from 1 January 2013 to 31 December 2015.
Study population
We included all patients diagnosed with TB irrespective of their age and HIV status in the study. Further, we registered patients that were in the TB register in 2012 and died in 2013. We also included as well as those that died or survived during the treatment period. Patients with unknown outcome were from the study or those with incomplete record were excluded. Basic demographic information including the age, gender, co-morbidities, tobacco Use, alcohol Use, substance use and duration on treatment were collected.
Study setting
The Ugu district is located in the rural KwaZulu-Natal province (Figure 1). Ugu district has a population of 733 228 people [37]. During the study period, Ugu district had the highest HIV prevalence and TB incidence of any district in KZN, 41.7% and 1096 per 100,000 people, respectively [37]. In terms of infectious TB (pulmonary smear-positive), Ugu ranks 12th, with 325 cases per 100,000 people, which is higher than the country's average of 208 cases per 100,000 people [37]. Elim clinic, a primary health

Powered by Editorial Manager® and ProduXion Manager® from Aries Systems Corporation

care facility was selected based on convenience, the study goal and the availability of information on HIV and TB infections".

Comment 16: this is about uGu- what about the specific area of your study- what is catchment population, what is outpatient headcount? what proportion or incidence of TB per year over the study period?
Response: In track change document on pages 7 and lines 136-142. Thanks, we have stated the following: "Ugu district has a population of 733 228 people [37]. During the study period, Ugu district had the highest HIV prevalence and TB incidence of any district in KZN, 41.7% and 1096 per 100,000 people, respectively [37]. In terms of infectious TB (pulmonary smear-positive), Ugu ranks 12th, with 325 cases per 100,000 people, which is higher than the country's average of 208 cases per 100,000 people [37]. Elim clinic, a primary health care facility was selected based on convenience, the study goal and the availability of information on HIV and TB infections".

Comment 17: was provincial authorization obtained? I am confused by the term Director of Health as no such position exists in the organogram.
Response: Thanks for this comment. Authorisation to access clinic records was granted by the Ugu Health District Manager. Apologies for the incorrect use of word 'Director'.

Comment 18: it would be good to also provide the socio-demographic profile of patients first before the HIV status of patients.
Response: In track change document on page 12and line 240. Thanks, before the HIV status table (Table 2), we have provided a socio-demographic table (Table 1).

Comment 19: p values determination- and if any of the information is statistically significant
Response: In track change document on page 15, table 3, line 277. Thanks, p-values have been provided. Duration on treatment and age-group were significant predictors of cure.

Comment 20: were the values statistically significant?
Response: In track change document on page 15, line 277. "The 95% CI shows that the values obtained were statistically significant".

Comment 21: to evaluate TB outcomes in HIV positive patients in rural primary healthcare in South Africa
Response: In track change document on page 17, lines 300. Thanks, it now reads: "This study evaluated the effect of HIV status and antiretroviral treatment on the treatment outcome of TB patients in a primary healthcare facility in rural South Africa".

Comment 22: what about findings from your analytical study?
Response: In track change document on page 17, lines 312-317. We have reported the findings from the analytical study as follows: "Longer treatment periods were associated with a lower risk of death in both the bivariate and covariate log-binomial regression models. Furthermore, in bivariate and covariate analysis, younger ages had a lower likelihood of being cured than older ages. However, HIV positive status of a TB patient had no effect on the likelihood of TB cure when compared to HIV negative status of a TB patient".

Comment 23: please clarify this- is this initiation of treatment or treatment duration
Response: In track change document on page 17, lines 315-317. It now reads "However, HIV positive status of a TB patient had no effect on the likelihood of TB cure when compared to HIV negative status of a TB patient".

Comment 24: how does this study relate to your findings. what is the plausible explanation for the similarities and differences?
Response: In track change document on page 18, lines 329-340. Thanks, the plausible explanation for the similarities and differences is stated as follows: "The similarities and differences between studies could be explained by ART uptake percentage and TB diagnosis. Following a review of these studies, the findings of our study was consistent with the study conducted in Kenya, owing to the ongoing scale-up and uptake of ART programs in both South Africa and Kenya. Table 2 of our study

revealed an ART uptake rate of 89.9%, compared to 61% in Kenya [42]. In contrast, 3.7% of ART uptake was recorded in Ghana [40] and 16% patients with extrapulmonary TB did not receive ART in Tanzania [41]. Aside from that, our study found 70.0% of patients to have pulmonary TB, compared to 36.9% in a Tanzanian study [41]. In fact, pulmonary TB is easier to diagnose than other types of TB. Previous data shows that “Extrapulmonary TB is associated with poor TB outcome in HIV-infected people [43]”.

Comment 25: is better adherence to treatment the only plausible explanation? if you review the data. It is noticed that age > 50 years also had a very high likelihood of mortality and therefore, i question your statement provided.
Response: In track change document on page 19, lines 349-353. Thanks, we have provided the following statement:” Another study has shown that older ages are more likely to develop extra-pulmonary and atypical forms of TB disease that are often harder to diagnose than conventional sputum smear-positive pulmonary tuberculosis [45]. Extra pulmonary and smear negative PTB were associated with high TB mortality [43, 46, 47].”

Comment 26: what is the plausible explanation for tobacco and TB treatment outcomes?
Response: In track change document on page 19, lines 362-366. The discussion part is improved as follows: “Smoking tobacco affects both innate and adaptive immunity, weakening the immunological defensive system in humans [50]. This appears to be the reason why smokers are at a higher risk of developing extra-pulmonary tuberculosis [51]. Apart from this, smoking tobacco increases the risk of mycobacterium TB infection as well as the development of tuberculosis in infected individuals [52]”.

Comment 27: please discuss the study limitations
Response: In track change document on page 20, lines 381-388. We have included the study strengths and limitations. It now read: “The strength of our study is our sample size which was representative of the study population thereby minimizing selection bias. This is substantial to estimate HIV/TB outcomes in rural settings. Our study has several limitations that could lead to underestimation HIV/TB outcomes. Retrospective cohort design uses records that have already been collected and we did not obtain the information on treatment completed outcome, HIV viral load and CD4 cells. As the fact, we were unable to accurately link all our patients to the various outcomes. It is also possible that some of the patients included in our study misclassified in the current analysis”.

Comment 28: this is a repetition of results without adequate considering for the overall aim and why the study was done?
Response: In track change document on page 21, lines 392-395, 400-404. Thanks, we have improved the conclusion as follows: “In conclusion, HIV positive status and antiretroviral treatment had no effect on the likelihood of TB cure in rural primary care when compared to HIV negative patients. However, the TB mortality rate in HIV positive patients was higher than in HIV negative patients”. And further recommendation: “TB mortality in rural SA. Furthermore, the TB success rate in rural SA may be lower than the WHO target. This study could have a significant impact on the HIV/TB program in rural SA. To achieve the End TB strategy in rural SA, various HIV/TB indicators should be reviewed, and gaps filled”.

Reviewer #2:
Comment 1: The abstract is well written. It contains the main findings and the conclusion of the study. The background is comprehensive and well written. Although, it does not reflect on the decreasing TB cases that South Africa is experiencing.
Response: In track change document on page 5-6, lines 103-108. Thanks, we have improved the background as follows:” TB incidence and mortality are declining in SA [7]. Data from a well-characterized rural SA population with high HIV prevalence and TB incidence demonstrated considerable spatial heterogeneity in people with recently-diagnosed TB and has shown that every percentage increase in ART coverage was associated with a 2% decrease in the odds of recently-diagnosed TB [23]”.

Comment 2: The methodology is clear, well defined. The paper does not clearly define treatment cure, treatment completed.

Powered by Editorial Manager® and ProduXion Manager® from Aries Systems Corporation

	Response: In track change document on pages 8, lines 160-167. We have referred to the WHO definitions. Cured: A pulmonary TB patient with bacteriologically confirmed TB at the beginning of treatment who was smear or culture negative in the last month of treatment and on at least one previous occasion. Treatment completed: A TB patient who completed treatment without evidence of failure but with no record to show that sputum smear or culture results in the last month of treatment and on at least one previous occasion were negative either because tests were not done or because results are unavailable [37].<br>Comment 3: The researcher used the outcome allocated by the facility. I am not sure whether these outcomes were checked. Sometimes patients who fulfil the cure criteria are captured as completed. It is also important to highlight that the outcomes are allocated mainly on the basis of negative TB smear microscopy. TB microscopy has a low sensitivity. TB culture is not done routinely in susceptible TB patients.<br>Response: In track change document on page 20, lines 384-388. Thanks, we have addressed this issue in the study weaknesses. As a retrospective cohort study, we conducted the records review of the patients. As a matter of fact, we were unable to accurately link all of our patients to the various outcomes due to missing data. It is also possible that some of the patients included in our study might have been misclassified in the current analysis.<br><br>Comment 5: My other issue is the choice of comparing cure or death. TB programme targets are based on treatment success rate (Cure and Completion). I agree that it is better to have a higher cure rate although for several reasons I have noted over the years the final sputum is often not collected hence the outcome will be "treatment completed".<br>Response: Thanks for the observation, as previously stated, retrospective cohort design used records that have already been collected and we did not obtain the information on treatment completed from the records.<br>Comment 6: Also, Table 1 indicates that 30 % of the cohort had a negative smear microscopy. Such patients may not have a "cure" outcome. Favorable outcomes include cure and completion rate. I am not sure what is the motivation for having cure, death, and other outcomes. It would have been useful to briefly explain why are there more treatment completion than treatment cure in this facility.<br>Response: In track change document on page 9, lines 160-163. Thanks, “cured” is defined as a pulmonary TB patient with bacteriologically confirmed TB at the beginning of treatment in an individual who was smear or culture negative in the last month of treatment and on at least one previous occasion. So, people may complete TB treatment without necessarily being cured. In this study we used these outcomes because they are key TB Program indicators as defined by the National TB Control Program. The focus of our study was to assess the effect of HIV status and antiretroviral treatment on TB treatment outcomes including cure. Our data has shown that HIV status and antiretroviral treatment did not have effect on TB cure but on TB mortality. We suggest that further studies are warranted to help explain low TB cure in this setting.<br>Comment 7: Data analysis is very clear except the issue of separating cure and completion. On page 11 of the manuscript, the Authors stated that among tobacco users the cure rate was for TB was 60 % and it was only 37 % among the non-tobacco users. In the conclusion, it is stated that the use of tobacco appeared to decrease the TB cure. It is also said that the cure rate was below district and provincial targets. To the best of my knowledge, there are no separate targets between cure and completion. Targets are based on Treatment Success Rate.<br>Response: Thanks for the observations, In track change document on page 19-20, Table 4. We separated cure and completion because they are two separate National TB Control Program treatment outcomes. The overall cure rate was 39.13% which falls below district and provincial targets based on South African District Health Barometer. Treatment Success Rate is the sum of cured and treatment completion rates.
**Additional Information:**	
**Question**	**Response**
**Financial Disclosure**<br><br>Enter a financial disclosure statement that describes the sources of funding for the	The author(s) received no specific funding for this work.

work included in this submission. Review the submission guidelines for detailed requirements. View published research articles from *PLOS ONE* for specific examples.<br><br>This statement is required for submission and **will appear in the published article** if the submission is accepted. Please make sure it is accurate.<br><br>**Unfunded studies**<br>Enter: *The author(s) received no specific funding for this work.*<br><br>**Funded studies**<br>Enter a statement with the following details:<br>• Initials of the authors who received each award<br>• Grant numbers awarded to each author<br>• The full name of each funder<br>• URL of each funder website<br>• Did the sponsors or funders play any role in the study design, data collection and analysis, decision to publish, or preparation of the manuscript?<br>• **NO** - Include this sentence at the end of your statement: *The funders had no role in study design, data collection and analysis, decision to publish, or preparation of the manuscript.*<br>• **YES** - Specify the role(s) played.<br><br>* typeset	
**Competing Interests**<br><br>Use the instructions below to enter a competing interest statement for this submission. On behalf of all authors, disclose any competing interests that could be perceived to bias this work—acknowledging all financial support and any other relevant financial or non-financial competing interests.<br><br>This statement is required for submission and **will appear in the published article** if the submission is accepted. Please make sure it is accurate and that any funding sources listed in your Funding Information	The authors have declared that no competing interests exist.

later in the submission form are also declared in your Financial Disclosure statement.<br><br>View published research articles from *PLOS ONE* for specific examples.<br><br>**NO authors have competing interests**<br><br>Enter: *The authors have declared that no competing interests exist.*<br><br>**Authors with competing interests**<br><br>Enter competing interest details beginning with this statement:<br><br>*I have read the journal's policy and the authors of this manuscript have the following competing interests: [insert competing interests here]*<br><br>* typeset	
**Ethics Statement**<br><br>Enter an ethics statement for this submission. This statement is required if the study involved:<br><br>• Human participants<br>• Human specimens or tissue<br>• Vertebrate animals or cephalopods<br>• Vertebrate embryos or tissues<br>• Field research<br><br>Write "N/A" if the submission does not require an ethics statement.<br><br>General guidance is provided below. Consult the submission guidelines for detailed instructions. **Make sure that all information entered here is included in the Methods section of the manuscript.**	The University of the Witwatersrand Human Research Ethics Committee approved the study. Authority to access medical records was obtained from the Director of Health, Ugu District. No attempt was made to link data to the individual identifier, and data was analysed anonymously.

Powered by Editorial Manager® and ProduXion Manager® from Aries Systems Corporation

**Format for specific study types**<br><br>**Human Subject Research (involving human participants and/or tissue)**<br>• Give the name of the institutional review board or ethics committee that approved the study<br>• Include the approval number and/or a statement indicating approval of this research<br>• Indicate the form of consent obtained (written/oral) or the reason that consent was not obtained (e.g. the data were analyzed anonymously)<br><br>**Animal Research (involving vertebrate animals, embryos or tissues)**<br>• Provide the name of the Institutional Animal Care and Use Committee (IACUC) or other relevant ethics board that reviewed the study protocol, and indicate whether they approved this research or granted a formal waiver of ethical approval<br>• Include an approval number if one was obtained<br>• If the study involved *non-human primates*, add *additional details* about animal welfare and steps taken to ameliorate suffering<br>• If anesthesia, euthanasia, or any kind of animal sacrifice is part of the study, include briefly which substances and/or methods were applied<br><br>**Field Research**<br><br>Include the following details if this study involves the collection of plant, animal, or other materials from a natural setting:<br>• Field permit number<br>• Name of the institution or relevant body that granted permission	
**Data Availability**<br><br>Authors are required to make all data underlying the findings described fully available, without restriction, and from the time of publication. PLOS allows rare exceptions to address legal and ethical concerns. See the PLOS Data Policy and FAQ for detailed information.	Yes - all data are fully available without restriction

A Data Availability Statement describing where the data can be found is required at submission. Your answers to this question constitute the Data Availability Statement and **will be published in the article**, if accepted.<br><br>**Important:** Stating 'data available on request from the author' is not sufficient. If your data are only available upon request, select 'No' for the first question and explain your exceptional situation in the text box.<br><br>Do the authors confirm that all data underlying the findings described in their manuscript are fully available without restriction?	
**Describe where the data may be found in full sentences. If you are copying our sample text, replace any instances of XXX with the appropriate details.**<br><br>• If the data are **held or will be held in a public repository**, include URLs, accession numbers or DOIs. If this information will only be available after acceptance, indicate this by ticking the box below. For example: *All XXX files are available from the XXX database (accession number(s) XXX, XXX.*).<br>• If the data are all contained **within the manuscript and/or Supporting Information files**, enter the following: *All relevant data are within the manuscript and its Supporting Information files.*<br>• If neither of these applies but you are able to provide **details of access elsewhere**, with or without limitations, please do so. For example:<br><br>*Data cannot be shared publicly because of [XXX]. Data are available from the XXX Institutional Data Access / Ethics Committee (contact via XXX) for researchers who meet the criteria for access to confidential data.*<br><br>*The data underlying the results presented in the study are available from (include the name of the third party*	All relevant data are within the manuscript and its Supporting Information files

Powered by Editorial Manager® and ProduXion Manager® from Aries Systems Corporation

and contact information or URL).<br>• This text is appropriate if the data are owned by a third party and authors do not have permission to share the data.<br><br>* typeset	
Additional data availability information:	

Powered by Editorial Manager® and ProduXion Manager® from Aries Systems Corporation

Manuscript

Click here to access/download;Manuscript;HIV_TB_co-infection_Clean_December 2_2021.docx

Effect of HIV status and antiretroviral treatment on treatment outcomes of tuberculosis patients in a rural primary healthcare clinic in South Africa

Peter S Nyasulu[1, 2], Emery Ngasama[2], Jacques L Tamuzi[1], Lovemore N Sigwadhi[1], Lovelyn U Ozougwu[3], Ruvimbo BC Nhandara[2], Birhanu T Ayele[1], Teye Umanah[4] Jabulani Ncayiyana[5]

Affiliations

[1]Division of Epidemiology and Biostatistics, Department of Global Health, Faculty of Medicine and Health Sciences, Stellenbosch University, Cape Town, South Africa

[2]Division of Epidemiology & Biostatistics, School of Public Health, Faculty of Health Sciences, University of the Witwatersrand, Johannesburg, South Africa

[3]Division of Public Health Surveillance and Response, National Institute for Communicable Diseases, Johannesburg, South Africa.

[4]Neuroscience Institute, Hackensack Meridian University at JFK Medical Center, New Jersey, United States of America.

[5]Public Health Medicine, School of Nursing and Public Health, University of KwaZulu-Natal, Durban, South Africa

Abstract

Background: Tuberculosis (TB) remains the leading cause of death among human immunodeficiency virus (HIV) infected individuals in South Africa. Despite the implementation of HIV/TB integration services at primary healthcare facility level, the effect of HIV on TB treatment outcomes has not been well investigated. To provide evidence base for TB treatment outcome improvement to meet End TB Strategy goal, we assessed the effect of HIV status on treatment outcomes of TB patients at a rural clinic in the Ugu Health District, South Africa.

Methods: We reviewed medical records involving a cohort of 508 TB patients registered for treatment between 1 January 2013 and 31 December 2015 at rural public sector clinic in KwaZulu-Natal province, South Africa. Data were extracted from National TB Programme clinic cards and the TB case registers routinely maintained at study sites. The effect of HIV status on TB treatment outcomes was determined by using multinomial logistic regression. Estimates used were relative risk ratio (RRR) at 95% confidence intervals (95%CI).

Results: A total of 506 patients were included in the analysis. Majority of the patients (88%) were new TB cases, 70% had pulmonary TB and 59% were co-infected with HIV. Most of HIV positive patients were on antiretroviral therapy (ART) (90% (n=268)). About 82% had successful treatment outcome (cured 39.1% (n=198) and completed treatment (42.9% (n=217)), 7% (n=39) died 0.6% (n=3) failed treatment, 3.9% (n=20) defaulted treatment and the rest (6.6% (n=33)) were transferred out of the facility. Using completed treatment as reference, HIV positive patients not on ART relative to

negative patients were more likely to have unsuccessful outcomes [RRR, 5.41; 95%CI, 2.11–13.86].

Conclusions: When compared HIV positive and HIV negative status, antiretroviral treatment had no effect on the likelihood of TB cure in rural primary care. The TB mortality rate in HIV positive patients, on the other hand, was higher than in HIV negative patients. Various HIV/TB indicators should be reviewed, and gaps filled to achieve the "End TB strategy" in rural South Africa.

Key words: TB; Treatment outcomes; co-infection; HIV Status, KwaZulu Natal province, South Africa.

1. Background

Tuberculosis (TB) is one of the major global infectious diseases with a high morbidity and mortality rate. Globally, an estimated 10.0 million (range 8.9–11.0 million) people fell ill with TB in 2019 [1]. There were 1.2 million (range, 1.1–1.3 million) TB deaths among human immunodeficiency virus (HIV) - negative people and an additional 208 000 (range, 177 000–242 000) TB deaths among HIV-positive people [1]. Ninety percent (90%) of all TB cases occur in adults, with a high prevalence in males compared to females [2]. The increase in incidence is also attributed to the development of multidrug resistant (MDR) and extremely drug resistant (XDR) strains of *Mycobacterium tuberculosis* [3]. Both MDR and XDR-TB are the causes of high TB mortality [4]. HIV prevalence in sub-Saharan Africa has significantly contributed to an increase in the incidence of TB [5]. An estimated 10 million people have been living with TB and 1.5 million have died in 2018 and the global burden of TB falls on 20 low- and middle-income (LMIC) countries, including sub-Saharan Africa [6]. The burden is attributed to the high rate of HIV/AIDS pandemics in those countries. Africa accounted for 84% of all TB/HIV deaths [6].

TB is the most common opportunistic infection and cause of death among PLWHIV in resource-limited countries [7, 8]. In the post-mortem period, the overall prevalence of TB was enormous and accounted for almost 40% of HIV-related facility-based deaths in resource-limited countries such as South Africa [9-11], Botswana [11, 12], Zimbabwe [11, 13, 14], Mozambique [11, 15], Uganda [11, 16] and Kenya [11, 17]. In contrast, the WHO reported 16% of HIV/TB related death in Africa [1].

South Africa (SA) ranked fifth among the highest TB incidences worldwide and first among TB/HIV co-infection cases with more than 65 percent of patients co-infected

with TB/HIV [5]. TB accounted for the third highest number of deaths in 2018 (6 %; n = 454,014), and combined TB and HIV accounted for 35.6% of all-cause mortality in SA [18-20]. The total number of people living with HIV in SA increased from an estimated 4.64 million in 2002 to 7.97 million by 2019 [21]. There are significant geographical variations in the rate of TB notification in SA which are not clearly correlated with the prevalence of HIV at the district level [21-23]. KwaZulu-Natal (KZN) carries the largest burden of HIV and related infections in SA, with HIV–TB co-infection estimated at approximately 70% [5, 24], and Ugu district reported 60.5% of TB/HIV co-infection rate [25].

According to recent data, 90% of people are aware of their HIV status of which 68% are on antiretroviral therapy (ART) and of which 87% are virally suppressed in SA [26]. ART may be associated with a reduced risk of HIV-associated TB disease in HIV-positive individuals due to a decrease in their viral load and an improvement in their immune system function [27]. On the one hand, while ART reduces new HIV infections, on the other hand, the marked decline in HIV-associated mortality has led to an increase in HIV prevalence and an increase in the number of life-years at TB risk. It is also plausible that HIV-positive people with TB infection may increase with CD4+ T-cell ART recovery, although the available data do not support this [27-29]. In addition, early initiation of ART during TB treatment (within 2–4 weeks) increased AIDS-free survival by 34–68% among patients with advanced HIV disease [11, 30-32]. Despite the inclusion of TB-HIV in the international and SA guidelines, the 2018 mortality rate for co-infected TB-HIV patients in SA was 73 (51-99) per 100,000 populations, which is more than three times higher than that for HIV-negative TB patients with a mortality rate of 37 (35–39) per 100,000 population [33]. TB incidence and mortality are declining in SA [7]. Data from a well-characterized rural SA

population with high HIV prevalence and TB incidence demonstrated considerable spatial heterogeneity in people with recently diagnosed TB, and has shown that every percentage increase in ART coverage was associated with a 2% decrease in the odds of recently diagnosed TB [23]

The End TB Strategy aims to reduce TB deaths by 90% and TB incidence rates by 80% in 2030 compared to 2015 [34]. Examining the various challenges that may impact on TB outcomes in rural primary health care in SA, the TB elimination target set for 2050 could be compromised if this dual burden of TB and HIV diseases is not controlled [5, 35]. The rate of TB incident stabilizes at a rate higher than that of the general population. These data highlight the need for more research into strategies for finding active cases in rural settings and the need to focus on strengthening primary health care [36]. Therefore, this retrospective study has been undertaken to evaluate TB outcomes in HIV positive patients in rural primary healthcare in Ugu Health District, KwaZulu-Natal, SA.

2. Methods

2.1. Study design

This retrospective cohort study of TB patients initiated on TB treatment was conducted from 1 January 2013 to 31 December 2015.

2.2 Study population

We included all patients diagnosed with TB irrespective of their age and HIV status in the study. Further, we included individuals that were registered as TB patients in 2012 and completed treatment or died in 2013. We also included as well as those that died or survived during the treatment period. Patients with unknown outcome or those with incomplete record were excluded Basic demographic information including the age, gender, co-morbidities, tobacco Use, alcohol Use, substance use and duration on treatment were collected.

2.3 Study setting

The Ugu Health District is in the rural aspects of the KwaZulu-Natal (KZN) province (Figure 1). Ugu district has a population of 733 228 people [37]. During the study period, Ugu district had the highest HIV prevalence and TB incidence of any district in KZN, 41.7% and 1096 per 100,000 people, respectively [37]. In terms of infectious TB (pulmonary smear-positive), Ugu ranks 12th, with 325 cases per 100,000 people, which is higher than the country's average of 208 cases per 100,000 people [37]. Elim clinic, a primary health care facility was selected based on convenience, the study goal and the availability of information on HIV and TB infections".

2.4. Sample size

A total of 478 cases were estimated to be adequate sample that would have the power to detect a significant difference in mortality between HIV positive and negative TB patients on anti-tuberculosis treatment. We used a 95% precision of estimate with power of 80% and estimated risk difference of 6% in outcome between exposed and unexposed.

2.5. Participant selection and definitions

All TB patients of all ages recorded in the TB treatment register, with HIV positive status and started on treatment were included in this study. These were all TB patients on treatment attending care at Elim Clinic, in Ugu District, KZN province within the stipulated time. At admission, patients were screened for other comorbidities including HIV infection. After following the treatment, the outcomes of treatment were recorded in seven mutually exclusive categories. Patients were classified as cured, completed treatment, interrupted treatment, moved, transferred out, failed, and died. The main interest of this study was to compare the likelihood of observing two main outcomes. These included being cured versus all other outcomes including death and the likelihood of dying versus staying alive regardless of the outcome of treatment. The World Health Organization (WHO) defines “cured” as follows: a pulmonary TB patient with bacteriologically confirmed TB at the start of treatment who was smear or culture negative in the last month of treatment and on at least one previous occasion, and "treatment completed": A TB patient who completed treatment without evidence of failure but with no record to show that sputum smear or culture results in the last month of treatment and on at least one previous occasion were negative either because tests were not done or because results are unavailable [38].

2.6 Outcome and exposures

The first outcome of interest was “cured” defined as 1 if the patient was declared “cured” by the healthcare team or 0 if otherwise. The second outcome was “death” coded 1 if the patient was declared dead and 0 if otherwise. Given the small number of deaths which were 35 in total, we created a nominal outcome, which captured all outcomes and combined them into 3 categories: cured, dead and other outcomes.

2.7. Data collection

Data were extracted from the TB register using structured data collection form from all the TB patients initiated on treatment at this rural public health clinic from 1 January 2013 and 31 December 2015. The data consists of collecting socio-demographic factors (age and gender), TB related factors (categories, pre-treatment smear, type, site, outcome duration, regimen at the baseline) and other factors such as ART initiation and co-trimoxazole prophylaxis, comorbidities, tobacco, and alcohol use. A data dictionary was formulated to guide data cleaning and analysis. No personal identifiers were extracted from the manual medical records.

2.8. Data management

Data were entered on excel spread sheet and imported into Stata software. This step was followed by thorough checking to assess missing values. Data were coded into numerical values and continuous data categorised; clean data were imported into Stata version 15 for analysis.

2.9. Data Analysis

The main interest of this study was to compare the likelihood of observing two main outcomes. These included being cured versus all other outcomes including death and the likelihood of dying versus staying alive regardless of the outcome of treatment. Data were summarized and presented in tables. As part of the descriptive statistics for

the population under study, summary tables were constructed to describe variables in terms of their frequency distribution. Pearson Chi-square and Fisher Exact tests were used to test association between independent variables and TB outcomes (cured, completed treatment, interrupted treatment, moved, transferred out, failed, and died). Given the fact that cured was a common outcome (39%), we fitted a log-binomial regression model to estimate the relative risk of being declared cured versus not being cured (Table 3). Finally, we fitted a multinomial logistic regression model predicting the likelihood of cured and death versus other outcomes. This allowed us to make some predictions on death, as a function of the selected socio-demographic covariates. Odds ratios were computed to assess the association between HIV status and TB mortality. Precision of estimate of the odds ratio was set at 5% significance level. All factors with $p \leq 0.1$ on univariate analysis were further analysed in multiple logistic regression model.

2.5. Ethical considerations

Ethical approval was granted by the Monash University Human Research Ethics Committee and permission to conduct the study and access the medical records at Elim Primary Health Care Clinic was obtained from the District Manager of Ugu Health District, KwaZulu Natal province. Waiver of consent was applied for and obtained since the data were retrieved from older clinic records. Data entry and analysis were done anonymously, and no attempt was made to link data to the individual identifier.

3. Results

Overall, the study included 508 patients. Of this total, 2 patients were dropped from further analysis because one was transferred to another facility the same day of admission to the hospital and the other one was on prophylaxis TMP-SMX and was

HIV negative. Hence, our final analytical sample included 506 patients for whom we had valid data on the TB treatment outcomes.

Table 1 reported the demographic characteristics and TB outcomes. The findings revealed that 39.13% (n=198) were reported as being cured at the end of treatment. The remaining 60.87% patients were classified as dead or alive, but not cured. Some patients were transferred to other facilities, whereas other were classified as treatment interrupted, transferred to another district, treatment outcome unknown, treatment completed and moved to another facility in the same district. Table 1 shows significant differences with regards to TB cured in terms of age, tobacco, and alcohol uses. For instance, younger patients had lower proportion of TB cure (22.62% of those less than 20 years of age) compared to older patients. Among tobacco users, we found that about 60% were TB cured, whereas among non-tobacco users only 37% were cured. Likewise, the findings estimated that alcohol users, had higher proportion of being TB cured (57.14% of all alcohol users) while non-alcohol users' patients only (38.08%) were cured.

The overall TB related mortality rate was 6.92% (n=35 patients). The mortality rate was significantly associated with HIV status. For instance, the mortality rate was higher among HIV positive patients (9.67%, i.e., 29 patients out of 300 patients) compared to HIV negative patients among whom only for 2.91% was reported (6 patients out of 206 HIV negative patients). Given the small sample size for the mortality rate, this was impractical to perform further analysis and obtain reliable estimates.

Table 1. Characteristics of TB patients presenting to Elim Clinic on the basis of TB cured outcomes

Factors	Not Cured n (%)	Cured n (%)	P-value	Alive) n (%)	Dead n (%)	p-value
	**308 (60.87)**	**198 (39.13)**		**471 (93.08)**	**35 (6.92)**	
**Duration on treatment[1]**	5.55 (0.16)	6.15 (0.13)	0.009*	6.01 (0.11)	2.74 (0.44)	0.001*
**HIV Status**						
Negative	123 (59.71)	83 (40.29)	0.658	200 (97.09)	6 (2.91)	0.004*
Positive	185 (61.67)	115 (38.33)		271 (90.33)	29 (9.67)	
**Age-group**						
<20	65 (77.38)	19 (22.62)	0.018*	82 (97.62)	2 (2.38)	0.101^F
20 – 29	78 (58.21)	56 (41.79)		124 (92.54)	10 (7.46)	
30 – 39	72 (55.38)	58 (44.62)		120 (92.31)	10 (7.69)	
40 – 49	46 (58.97)	32 (41.03)		75 (96.15)	3 (3.85)	
50+	47 (58.75)	33 (41.25)		70 (87.50)	10 (12.50)	
**Sex**						
Female	138 (61.61)	86 (38.39)	0.762	205 (91.52)	19 (8.48)	0.216
Male	170 (60.28)	112 (39.72)		266 (94.33)	16 (5.67)	
**Comorbidity**						
No	286 (60.98)	183 (39.02)	0.855	438 (93.39)	31 (6.61)	0.311^F
Yes	22 (59.46)	15 (40.54)		33 (89.19)	4 (10.81)	
**Tobacco Use**						
No	292 (62.66)	174 (37.34)	0.005*	432 (92.70)	34 (7.30)	0.345^F
Yes	16 (40.00)	24 (60.00)		39 (97.50)	1 (2.50)	
**Alcohol Use**						
No	296 (61.92)	182 (38.08)	0.045*	443 (92.68)	35 (7.32)	0.246^F
Yes	12 (42.86)	16 (57.14)		28 (100.00)	0 (0.00)	
**Substance Use**						
No	292 (60.08)	194 (39.92)	0.101	452 (93.00)	34 (7.00)	1.00^F
Yes	16 (80.00)	4 (20.00)		19 (95.00)	1 (5.00)	

Note: [1] Duration of treatment in months

All p-value from Pearson Chi-square test, unless otherwise determined.

** p-value < 0.05; ^{F}indicates results from Fisher Exact test*

Table 2: Characteristics of TB patients presenting to Elim Clinic on the basis of HIV status.

Characteristics	HIV Negative n(%) 178 (37.2)	HIV Positive n(%) 300 (62.8)	P-value
**Categories of TB**			
New	161 (91.0)	259 (86.3)	0.395¶
RAC	8 (4.5)	25 (8.3)	
RF	5 (2.8)	8 (2.7)	
RI	3 (1.7)	8 (2.7)	
**Pre-treatment sputum smear status**			
Negative	27 (29.7)	41 (30.4)	0.910‡
Positive	64 (70.3)	94 (69.6)	
**Type of Tuberculosis**			
Pulmonary	124 (69.7)	210 (70.0)	0.938‡
Extra-Pulmonary	54 (30.3)	90 (30.0)	
**Site of Tuberculosis**			
			0.047*¶
Bones/Joints	1 (2.0)	0 (0.0)	
Lymph Nodes	0 (0.0)	4 (4.5)	
Miliary	4 (7.8)	6 (6.7)	
Meningitis	2 (3.9)	17 (19.1)	
Primary	12 (23.5)	17 (19.1)	
Pleura	12 (23.5)	22 (24.7)	
Other organs	20 (39.2)	23 (25.8)	
**Receiving ART**			
No	172 (100.0)	30 (10.1)	<0.001*¶
Yes	0 (0.0)	267 (89.9)	
**Co-trimoxazole Prophylaxis**			
No	173 (100.0)	22 (7.3)	<0.001*¶
Yes	0 (0.0)	278 (92.7)	
**Outcome duration(days)**	184 (176-203)**	184 (173-209)*	0.7547†
**Comorbidity**			
No	155 (87.1)	286 (95.3)	0.001*
Yes	23 (12.9)	14 (4.7)	
**Regimen type at baseline**			
HRZE	157 (88.7)	280 (93.4)	0.005*¶
HR	15 (8.5)	10 (3.3)	
RHZ	5 (2.8)	3 (1.0)	
HRZES	0 (0.0)	7 (2.3)	

N- Total number in each group; % - row percentages; Numbers may not add-up because of missing variables.

*Significant p-value;

**Median and Inter-quartile ranges (IQR); Test statistic based on ¶Fisher's Exact, ‡Chi-square and Wilcoxson †Rank-sum test.

HIV – Human Immunodeficiency Virus; ART – Antiretroviral therapy; Categories of TB treatment: RAC – retreatment after completion of a previous course without microscopic result; RF – Treatment failure: a patient who, while on treatment, remained or became again smear positive five months or later after commencing treatment; RI – Treatment after interruption: a patient whose treatment is interrupted for two or more months and who returned to the health service.

HRZE – Isoniazid, Rifampicin, Pyrazinamide and Ethambutol for New adult patients; HRZ – Isoniazid, Rifampicin and Pyrazinamide for New Paediatric patients; HRZES – Isoniazid, Rifampicin, Pyrazinamide, Ethambutol and Streptomycin for Retreatment patients.

Table 3 below presented the results from the univariate and multivariate log-binomial model, which sought to identify covariates associated with cure event. These factors included ‘duration of treatment, age, comorbidity, tobacco, alcohol use, and HIV positive status’ which was our main exposure of interest

We found that HIV positive status showed to affect the likelihood of TB cure among patients on TB treatment. The results seem to suggest that HIV positive patients were 12% less likely to be cured from TB compared to the HIV negative patients, however this finding was not statistically significant (RR: 0.88; 95% C.I.: 0.69 – 1.11). The length of TB treatment was associated with TB cure. The results show that for each additional month on TB treatment there was a 3% significantly increased likelihood of TB cure (RR: 1.03; 95% C.I.: 1.01 – 1.06). Alcohol use and Tobacco smoking were not significantly associated with increased risk of cure.

Table 3. Factors associated with cure among TB patients presenting for care at Elim Clinic

Factors	Univariate Analysis RR (95% C.I.)	Adjusted Analysis RR (95% C.I.)	p-value
**Duration on treatment**	**1.02 (1.01 – 1.05)**	**1.03(1.01 – 1.06)**	0.039
**HIV Status**			
Negative	Ref	Ref	
Positive	0.95 (0.76-1.19)	0.88(0.69-1.11)	0.124
**Age-group**			
<20	Ref	Ref	
20 – 29	1.85(1.19 – 2.88)	1.82(1.15 – 2.91)	0.014
30 – 39	1.97(1.27 -3.06)	2.00(1.26 – 3.17)	0.008
40 – 49	1.81(1.13 – 2.92)	1.79(1.10 – 2.91)	0.033
50+	1.82(1.13 – 2.93)	1.77(1.09 – 2.85)	0.036
**Sex**			
Female	Ref	-	
Male	1.03(0.83 – 1.29)	-	
**Comorbidity**			
No	Ref	-	
Yes	1.04(0.69 – 1.56)	-	
**Tobacco Use**			
No	Ref	Ref	
Yes	1.60(1.22 – 2.12)	1.54(0.95 – 2.48)	0.117
**Alcohol Use**			
No	Ref	Ref	
Yes	1.5(1.07 – 2.11)	0.93(0.51 – 1.69)	0.909

Table 4 below present's results from the multinomial logistic regression model with two levels of outcomes 'dead and other'. We found that HIV status and age were associated with increased mortality among TB patients on treatment. The results shows that HIV positive TB patients were nearly 4 times likely to die of TB compared to the HIV negative patients, and this finding was statistically significant (RRR: 3.73; 95% C.I.: 1.24 – 11.19). Furthermore, older age was associated with 5 times increased risk of dying among TB patients aged 50 years and above, however this was not statistically significant (RR: 4.99; 95% C.I. 0.88-28.50). In addition, duration of TB

treatment was associated with reduced risk of dying. We found that for each additional month on TB treatment there was a 44% significant reduction in the risk of TB mortality (RR: 0.56; 95% C.I.: 0.47 – 0.66).

Table 4. Risk factors associated with death among TB patients presenting for care at Elim Clinic

Factors	Univariate Analysis RRR (95% C.I.)	Adjusted Analysis RRR (95% C.I.)
**Duration on treatment**	0.54 (0.46 – 1.13)	0.56 (0.47 – 0.66)
**HIV Status**		
Negative	Ref	Ref
Positive	3.63 (1.46 – 9.02)	3.73 (1.24 – 11.19)
**Age group**		
<20	Ref	Ref
20 – 29	4.63 (0.98 – 21.97)	2.40 (0.45 – 12.78)
30 – 39	5.08 (1.07 – 24.13)	2.18 (0.39 – 12.29)
40 – 49	2.20 (0.35 – 13.71)	1.46 (0.20 – 10.83)
50+	8.51 (1.77 – 40.98)	4.99 (0.88 – 28.50)
**Sex**		
Female	Ref	Ref
Male	0.65 (0.32 – 1.32)	0.61 (0.26 – 1.46)
**Co-morbidity**		
No	Ref	Ref
Yes	1.83 (0.58 – 5.75)	1.30 (0.29 – 5.80)
**Tobacco Use**		
No	Ref	Ref
Yes	0.51 (0.06 – 3.95)	0.56 (0.06 – 4.96)

Other outcomes include…. Alive, transferred, defaulted etc

4. Discussion

This study evaluate[illegible]he effect of HIV status and antiretroviral treatment on the treatment outcome of TB patients in a primary healthcare facility in rural South Africa. The study comprised of 282 males (55.7) and 224 females (44.3) with a median age of 32 (IQR: 23-43) years. The main outcomes of the study were categorized as cured or dead or other. This study found that 39.13% (198/506) of the patients were cured of Tuberculosis, of which 115 were HIV positive. Even though the patient's records did not clarify that TB patients completed a full course of treatment, our findings shows that the treatment success rate may be estimated as below the WHO target of 90% [39]. The overall TB related mortality rate was 6.92% (n=35 patients) and the study showed that the risk of mortality rate was significantly associated with HIV status. For instance, the mortality rate was higher among HIV positive patients (9.67%, i.e., 29 patients out of 300 patients) compared to HIV negative patients (2.91%, 6 patients out of 206 HIV negative patients). Longer treatment period was associated with a lowered risk of death; however, HIV positive status of a TB patient had no effect on the likelihood of TB cure when compared to HIV negative status of a TB patient.

In reviewing other studies conducted in rural Africa, HIV status did not appear to have any effect on successful treatment of TB, and this could be due to lack of sufficient data in a study conducted in Ghana [40], however, the same study showed that HIV status was associated with deat[illegible]0]. Our study found a close association between HIV-positive status and increased risk of dying of TB like the Ghana study that showed a higher mortality rate among HIV/TB co-infected individuals [40]. Another study in Tanzania found an EPTB prevalence of 5.6% with a high mortality rate of 14.3% and a combined death/LTFU rate of 46.8% among people living with HIV [41]. Patients with EPTB not receiving ART and >45 years of age were at a higher risk for poor outcomes

[41]. There were no differences in treatment success rates for HIV-positive TB patients on ART and HIV-negative in Kenya [42]. The similarities and differences between studies could be explained by the levels of ART uptake and TB diagnosis. Following a review several of these studies, our study finding is consistent with the study conducted in Kenya, owing to the ongoing increasing scale-up and uptake of ART among HIV positive individuals in both South Africa and Kenya. In Table 2 we show an ART uptake rate of 89.9%, compared to 61% in Kenya [42]. In contrast, 3.7% of ART uptake was recorded in Ghana [40] and 16% patients with extrapulmonary TB did not receive ART in Tanzania [41]. Aside from that, our study found 70.0% of patients to have pulmonary TB, compared to 36.9% in a Tanzanian study [41]. In fact, pulmonary TB is easier to diagnose than other types of TB. Previous data shows that Extrapulmonary TB is associated with poor TB outcome in HIV-infected people [43]. In our study low number of people were on ART at that time as the scaling up program was still in its infancy stage, hence difficult to make a meaningful analysis to assess its impact on TB outcome.

Age was reported as significantly associated with being cured. In our study, older patients were less likely to be cured and more likely to die of TB compared to younger patients. These results are in line with the study conducted in Zimbabwe where elderly patients had a higher risk of not being cured and high risk of dying [44]. Another study reported that older age patients are more likely to develop extra-pulmonary and atypical forms of TB disease that are often harder to diagnose than sputum smear-positive pulmonary tuberculosis [45]. Extra pulmonary and smear negative PTB have all previously shown to be associated with high TB mortality [43, 46, 47].

Existing evidence from previous meta-analysis of high-quality studies has shown that smoking tobacco was noted to influence the outcome of TB treatment as smokers had

a likelihood of being cured compared with non-smokers (OR of 2.6 (95%CI 2.1–3.4) [48]. Further, the associations between smoking and TB mortality showed a pooled OR of 1.3 (95%CI 1.1–1.6) [48]. Another large systematic review showed that cigarette smoking was significantly linked with poor TB treatment outcomes [49]. This shows that evidence on the effect of smoking on TB outcomes remains inconclusive. The findings of our study however confirm what has been previously reported showing an association between smoking tobacco and TB cure. Smoking tobacco affects both innate and adaptive immunity, weakening the immunological defensive system in humans [50]. This appears to be the reason why smokers are at a higher risk of developing extra-pulmonary tuberculosis [51]. Apart from this, smoking tobacco increases the risk of mycobacterium TB infection as well as the development of tuberculosis in infected individuals [52]. However, it does not have a negative effect on cure among those on TB treatment.

A recent study in the KZN revealed that staff rotation challenges across different services have been contributed to skills insufficiency (80%); staff shortage (50%); high staff turnover, absenteeism, and staff personal preferences (30%) in the KZN [5]. Despite a substantial investment in the clinical expansion program in SA, sparsely populated rural areas are still constrained in geographic distribution and access to health services (seen at a lower PHC utilization rate) and this is a major challenge in provision HIV and TB services in rural areas [33]. In this context, integrated services could enhance and promote equity as they maximize the potential benefit for each facility visit for health care access [33]. Besides this, a large TB cohort in Western Cape, South Africa showed that concomitant diseases were not associated with TB mortality [53]. This has been clearly shown in our study. However, in the same study,

factors such as ‘treatment category’ and ‘comorbidity’ were significantly associated with unsuccessful TB treatment [53].

The strength of our study is our sample size and its representativeness of the rural population, thereby minimizing selection bias. It was sufficient to estimate HIV/TB outcomes in rural primary care settings. Our study has several limitations that could lead to underestimation of HIV/TB outcomes. Retrospective cohort design uses records that have already been collected. This is an inherent weakness of the nature of use of existing medical records as data used is dependent on data documented in the records. We did not obtain the information on treatment completed outcome, HIV viral load and CD4 cells as this information was not available in the patients’ medical records. As such, we were unable to accurately link all our patients’ outcomes to these specific exposures. It is also possible that some of the patients included in our study were misclassified based on exposure or outcome status during analysis.

5. Conclusion

6. In conclusion, HIV positive status, and length of treatment were significantly associated with TB treatment outcomes in this rural primary care facility. The risk of dying was higher among the HIV positive TB patients while duration on treatment was associated with increased likelihood of cure and reduced risk of mortality. Despite a higher ART coverage in general, HIV status was a strong predictor of the TB mortality in rural SA. However, the TB treatment success rate in rural SA was lower than the WHO target. This study could have a significant impact on the reinforcement of HIV/TB program integration in rural SA. To achieve the End TB strategy in rural SA, HIV/TB integration services needs to be reviewed to identify potential challenges in its implementation. Furthermore, various HIV/TB indicators should be reviewed to allow for strengthening of TB/HIV integration monitoring approach by the TB program. **Supporting information**

S1 Data analysis commands

7. Acknowledgements: The authors would like to thank Ugu Health District management for the permission to conduct the study and the staff of Elim Clinic for the support provided during the duration of this study.

8. Author Contributions

Conceptualization: Peter S Nyasulu, Jabulani Ncayiyana

Data curation: Peter S Nyasulu, Lovemore N Sigwadhi, Emery Ngasama, Teye Umanah, Jabulani Ncayiyana

Formal analysis: Lovemore N Sigwadhi, Emery Ngasama, Lovelyn U Ozougwu, Ruvimbo BC Nhandara, Teye Umanah, Birhanu T Ayele

Investigation: Peter S Nyasulu, Emery Ngasama, Jacques L Tamuzi, Birhanu T Ayele, Jabulani Ncayiyana.

Methodology: Peter S Nyasulu, Emery Ngasama, Jacques L Tamuzi, Lovemore N Sigwadi, Birhanu T Ayele, Jabulani Ncayiyana.

Supervision: Peter S Nyasulu, Birhanu T Ayele, Jabulani Ncayiyana

Writing – original draft: Peter S Nyasulu, Jacques L Tamuzi, Lovelyn U Ozougwu Ruvimbo BC Nhandara

Writing – review & editing: Peter S Nyasulu, Emery Ngasama, Jacques L Tamuzi, Lovemore N Sigwadhi, Lovelyn U Ozougwu, Ruvimbo BC Nhandara, Birhanu T Ayele, Teye Umanah, Jabulani Ncayiyana.

Financial Disclosure: The author(s) received no specific funding for this work.

Competing Interests: The authors have declared that no competing interests exist.

Ethics Statement: Ethical approval to conduct the study was obtained from the Monash University Research Ethics Committee.

Data Availability: Dataset are available upon request by submitting the request form to the corresponding author (email: pnyasulu@sun.ac.za).

9. References

1. World Health Organization. Global tuberculosis report, 2020. Available from: https://apps.who.int/iris/bitstream/handle/10665/336069/9789240013131-eng.pdf.
2. Department of Health (Republic of South Africa). National Tuberculosis Management Guidelines, 2009. Available from: https://www.tbonline.info/media/uploads/documents/south_african_national_tuberculosis_management_guidelines_%282009%29.pdf
3. Roy E, Lowrie DB, Jolles SR. Current strategies in TB immunotherapy. Curr Mol Med. 2007;7(4):373-86.
4. Alemu A, Bitew ZW, Worku T, Gamtesa DF, Alebel A. Predictors of mortality in patients with drug-resistant tuberculosis: A systematic review and meta-analysis. PLoS One. 2021;16(6): e0253848.
5. Kalonji D, Mahomed OH. Health system challenges affecting HIV and tuberculosis integration at primary healthcare clinics in Durban, South Africa. Afr J Prim Health Care Fam Med. 2019;11(1): e1-e7.
6. World Health Organization. HIV-Associated Tuberculosis, 2019. Available from: https://www.who.int/tb/areas-of-work/tb-hiv/tbhiv_factsheet.pdf?ua=1.
7. Naidoo K, Gengiah S, Yende-Zuma N, Padayatchi N, Barker P, Nunn A, Subrayen P, Abdool Karim SS. Addressing challenges in scaling up TB and HIV treatment integration in rural primary healthcare clinics in South Africa (SUTHI): a cluster randomized controlled trial protocol. Implement Sci. 2017;12(1):129.
8. Manosuthi W, Wiboonchutikul S, Sungkanuparph S. Integrated therapy for HIV and tuberculosis. AIDS Res Ther 2016; 13(1):1-12.

9. Rennert WP, Kilner D, Hale M, Stevens G, Stevens W, Crewe-Brown H. Tuberculosis in children dying with HIV-related lung disease: clinical-pathological correlations. Int J Tuberc Lung Dis 2002; 6:806–813.
10. Wong EB, Omar T, Setlhako GJ, Osih R, Feldman C, Murdoch DM, et al. Causes of death on antiretroviral therapy: a post-mortem study from South Africa. PLoS One 2012; 7: e47542.
11. Gupta Rishi K, Lucas Sebastian B, Fielding Katherine L, Lawn Stephen D. Prevalence of tuberculosis in post-mortem studies of HIV-infected adults and children in resource-limited settings: a systematic review and meta-analysis. AIDS (London, England) 2015; 29(15):1987-2002.
12. Ansari NA, Kombe AH, Kenyon TA, Hone NM, Tappero JW, Nyirenda ST, et al. Pathology and causes of death in a group of 128 predominantly HIV-positive patients in Botswana, 1997-1998. Int J Tuberc Lung Dis 2002; 6:55–63.
13. Ikeogu MO, Wolf B, Mathe S. Pulmonary manifestations in HIV seropositivity and malnutrition in Zimbabwe. Arch Dis Child 1997; 76:124–128.
14. Nathoo KJ, Gondo M, Gwanzura L, Mhlanga BR, Mavetera T, Mason PR, et al. Fatal Pneumocystis carinii pneumonia in HIV-seropositive infants in Harare, Zimbabwe. Trans R Soc Trop Med Hyg 2001; 95:37–39.
15. Carrilho C, Monteiro E, Ussene E, Macie A, Fernandes F, Lorenzoni C, et al. Causes of death in HIV/AIDS patients in Maputo Central Hospital: a retrospective study from 2011. Histopathology 2012; 61:1–2
16. Cox JA, Lukande RL, Nelson AM, Mayanja-Kizza H, Colebunders R, Van Marck E, et al. An autopsy study describing causes of death and comparing

clinico-pathological findings among hospitalized patients in Kampala. Uganda PLoS One 2012; 7: e33685.

17. Chakraborty R, Pulver A, Pulver LS, Musoke R, Palakudy T, D'Agostino A, et al. The postmortem pathology of HIV-1-infected African children. Ann Trop Paediatr 2002; 22:125–131.
18. Stats sa. TB tops leading causes of death in SA in 2018. Available at http://www.statssa.gov.za/?p=14435.
19. Pillay-van Wyk V, Bradshaw D. Mortality, and socioeconomic status: the vicious cycle between poverty and ill health. Lancet Glob Health. 2017;5(9): e851-e852.
20. Statistic South Africa. Mid-year population estimates, 2019. Available from: https://www.statssa.gov.za/publications/P0302/P03022019.pdf.
21. Nanoo A, Izu A, Ismail NA, Ihekweazu C, Abubakar I, Mametja D, Madhi SA. Nationwide and regional incidence of microbiologically confirmed pulmonary tuberculosis in South Africa, 2004-12: a time series analysis. Lancet Infect Dis. 2015;15(9):1066-1076.
22. Sloot R, Maarman GJ, Osman M, Marx FM. On Behalf of The Desmond Tutu TB Centre-Working Group Data Analysis and Modelling FM. Variation in HIV prevalence and the population-level effects of antiretroviral therapy in reducing tuberculosis incidence in South Africa. S Afr Med J.;108(8):12370.
23. Tomita A, Smith CM, Lessells RJ, Pym A, Grant AD, de Oliveira T, Tanser F. Space-time clustering of recently diagnosed tuberculosis and impact of ART scale-up: Evidence from an HIV hyper-endemic rural South African population. Sci Rep. 2019;9(1):10724.

24. Health System Trust. Durban. District Health Barometer 2015/16. Available at https://www.hst.org.za/publications/District%20Health%20Barometers/District%20Health%20Barometer%202015_16.pdf
25. Health Systems Trust. Tuberculosis, 2015. Available at https://www.hst.org.za/publications/District%20Health%20Barometers/9%20(Section%20A)%20Tuberculosis.pdf
26. UNAIDS. UNAIDS data 2019. Available at https://www.unaids.org/sites/default/files/media_asset/2019-UNAIDS-data_en.pdf
27. van Halsema CL, Fielding KL, Chihota VN, George EC, Lewis JJ, Churchyard GJ, Grant AD. Brief Report: The Effect of Antiretroviral Therapy and CD4 Count on Markers of Infectiousness in HIV-Associated Tuberculosis. J Acquir Immune Defic Syndr. 2015;70(1):104-8.
28. Khan PY, Crampin AC, Mzembe T, Koole O, Fielding KL, Kranzer K, Glynn JR. Does antiretroviral treatment increase the infectiousness of smear-positive pulmonary tuberculosis? Int J Tuberc Lung Dis. 2017;21(11):1147-1154.
29. Abdool Karim SS, Naidoo K, Grobler A, Padayatchi N, Baxter C, Gray AL, Gengiah T, Gengiah S, Naidoo A, Jithoo N, Nair G, El-Sadr WM, Friedland G, Abdool Karim Q. Integration of antiretroviral therapy with tuberculosis treatment. N Engl J Med. 2011;365(16):1492-501.
30. Blanc FX, Sok T, Laureillard D, Borand L, Rekacewicz C, Nerrienet E, et al. Earlier versus later start of antiretroviral therapy in HIV-infected adults with tuberculosis. New England Journal of Medicine 2011; 365(16):1471-81.

31. Havlir DV, Kendall MA, Ive P, Kumwenda J, Swindells S, Qasba SS, et al. Timing of antiretroviral therapy for HIV-1 infection and tuberculosis. New England Journal of Medicine 2011; 365(16):1482-91.

32. World Health Organization. WHO Global Tuberculosis Report — South Africa profile, 2019. Available from: file:///D:/Files/The%20burden%20of%20TB-COVID-19%20in%20Sub-Saharan%20Africa/2019%20WHO%20Global%20Tuberculosis%20Report%20-%20South%20Africa%20profile.pdf

33. Scott V, Sanders D. Evaluation of how integrated HIV and TB programs are implemented in South Africa and the implications for rural-urban equity 2013. Available from: http://repository.uwc.ac.za/handle/10566/1111

34. World Health Organization. Global tuberculosis report (executive summary), 2018. Available from: https://www.who.int/tb/publications/global_report/tb18_ExecSum_web_4Oct18.

35. Kwan CK, Ernst JD. HIV and tuberculosis: a deadly human syndemic. Clin MicrobiolRev 2011; 24(2):351-76.

36. Houlihan CF, Mutevedzi PC, Lessells RJ, Cooke GS, Tanser FC, Newell ML. The tuberculosis challenge in a rural South African HIV programme. BMC Infect Dis. 2010; 10:23.

37. Department of Health Province/KwaZulu Natal. District Health Plan 2015/2016. UGU KwaZulu-Natal, 2015. Available at http://www.kznhealth.gov.za/Strategic/DHP/2015-16/ethekwini.pdf

38. World Health Organization. Treatment of tuberculosis: guidelines, 2010. Available at

https://apps.who.int/iris/bitstream/handle/10665/44165/9789241547833_eng.pdf

39. World Health Organization. Global Tuberculosis 2018 report, 2018. Available at https://www.who.int/tb/publications/global_report/gtbr2018_main_text_28Feb2019.pdf
40. Ogyiri L, Lartey M, Ojewale O, Adjei AA, Kwara A, Adanu RM, Torpey K. Effect of HIV infection on TB treatment outcomes and time to mortality in two urban hospitals in Ghana-a retrospective cohort study. Pan Afr Med J. 2019; 32:206.
41. Arpagaus A, Franzeck FC, Sikalengo G, Ndege R, Mnzava D, Rohacek M, Hella J, Reither K, Battegay M, Glass TR, Paris DH, Bani F, Rajab ON, Weisser M; KIULARCO Study Group. Extrapulmonary tuberculosis in HIV-infected patients in rural Tanzania: The prospective Kilombero and Ulanga antiretroviral cohort. PloS One. 2020;15(3): e0229875.
42. Owiti P, Zachariah R, Bissell K, Kumar AM, Diero L, Carter EJ, Gardner A. Integrating tuberculosis and HIV services in rural Kenya: uptake and outcomes. Public Health Action. 2015;5(1):36-44.
43. Mabunda TE, Ramalivhana NJ, Dambisya YM. Mortality associated with tuberculosis/HIV co-infection among patients on TB treatment in the Limpopo province, South Africa. Afr Health Sci. 2014;14(4):849-54.
44. Ncube RT, Takarinda KC, Zishiri C, Van den Boogaard W, Mlilo N, Chiteve C, et al. Age-stratified tuberculosis treatment outcomes in Zimbabwe: are we paying attention to the most vulnerable? Public health action 2017;7(3):212-7.
45. Negin J, Abimbola S, Marais BJ. Tuberculosis among older adults—time to take notice. Int J Infect Dis. 2015; 32:135-7.

46. Rao VK, Iademarco EP, Fraser VJ, Kollef MH. The impact of comorbidity on mortality following in-hospital diagnosis of tuberculosis. Chest. 1998; 114:1244–1252.
47. Peter JG, Theron G, Singh N, Singh A, Dheda K. Sputum induction to aid diagnosis of smear-negative or sputum-scarce tuberculosis in adults in HIV-endemic settings. Eur Respir J. 2014;43(1):185-94.
48. Slama K, Chiang CY, Enarson DA, Hassmiller K, Fanning A, Gupta P, Ray C. Tobacco, and tuberculosis: a qualitative systematic review and meta-analysis. Int J Tuberc Lung Dis. 2007;11(10):1049-61.
49. Burusie A, Enquesilassie F, Addissie A, Dessalegn B, Lamaro T. Effect of smoking on tuberculosis treatment outcomes: A systematic review and meta-analysis. PLoS One. 2020;15(9): e0239333.
50. Qiu F, Liang C-L, Liu H, Zeng Y-Q, Hou S, Huang S, Lai X, et al. Impacts of cigarette smoking on immune responsiveness: Up and down or upside down? Oncotarget 2017, 8(1):268.
51. Maurya V, Vijayan VK, Shah A. Smoking and tuberculosis an association overlooked. The international journal of tuberculosis and lung disease: the official journal of the International Union against Tuberculosis and Lung Disease 2002, 6(11):942–951.
52. Bates MN, Khalakdina A, Pai M, Chang L, Lessa F, Smith KR. Risk of tuberculosis from exposure to tobacco smoke: a systematic review and meta-analysis. Archives of internal medicine 2007, 167(4):335–342.
53. Azeez A, Ndege J and Mutambayi R. Associated factors with unsuccessful tuberculosis treatment outcomes among tuberculosis/HIV coinfected patients

with drug-resistant tuberculosis. International Journal of Mycobacteriology 2018; 7(4): 347.

 Click here to access/download;Figure;Figure 1.docx

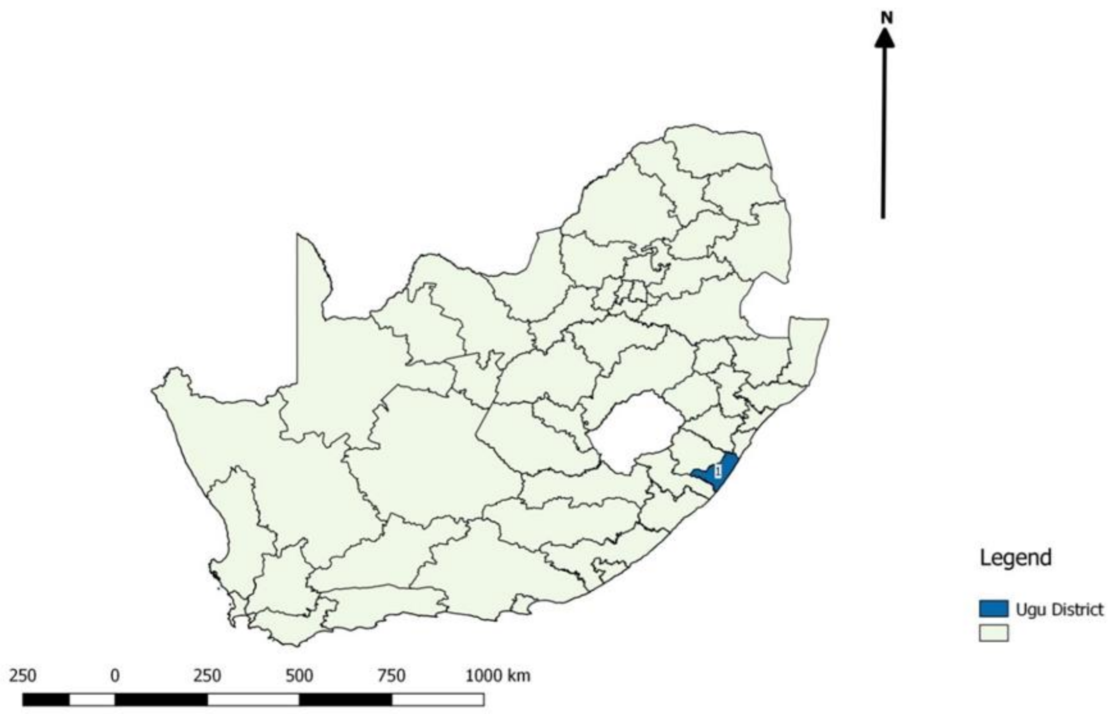

Figure 1: Map of study setting: Ugu district in KwaZulu Natal, South Africa

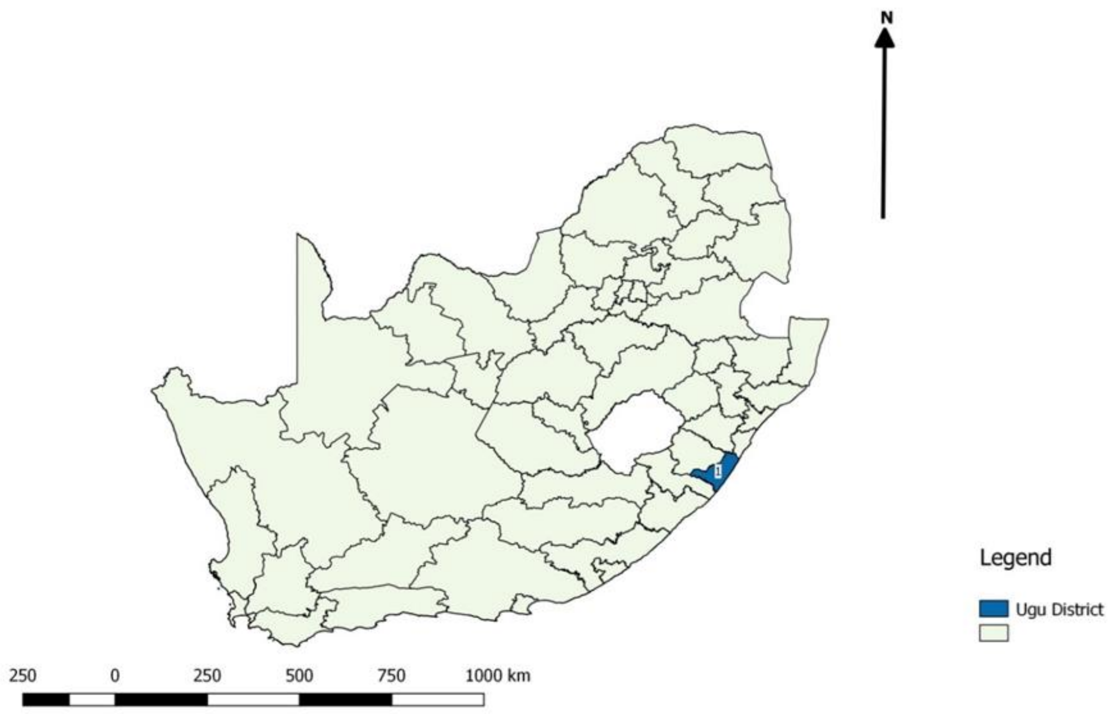

Figure 1: Map of study setting: Ugu district in KwaZulu Natal, South Africa

Click here to access/download
Supporting Information
Supporting Information_24_12_2022.xls

Effect of HIV status and antiretroviral treatment on treatment outcomes of tuberculosis patients in a rural primary healthcare clinic in South Africa

Peter S Nyasulu[1, 2], Emery Ngasama[2], Jacques L Tamuzi[1], Lovemore N Sigwadhi[1], Lovelyn U Ozougwu[3], Ruvimbo BC Nhandara[2], Birhanu T Ayele[1], Teye Umanah[4] Jabulani Ncayiyana[5]

Affiliations

[1]Division of Epidemiology and Biostatistics, Department of Global Health, Faculty of Medicine and Health Sciences, Stellenbosch University, Cape Town, South Africa

[2]Division of Epidemiology & Biostatistics, School of Public Health, Faculty of Health Sciences, University of the Witwatersrand, Johannesburg, South Africa

[3]Division of Public Health Surveillance and Response, National Institute for Communicable Diseases, Johannesburg, South Africa.

[4]Neuroscience Institute, Hackensack Meridian University at JFK Medical Center, New Jersey, United States of America.

[5]Public Health Medicine, School of Nursing and Public Health, University of KwaZulu-Natal, Durban, South Africa

Abstract

Background: Tuberculosis (TB) remains the leading cause of death among human immunodeficiency virus (HIV) infected individuals in South Africa. Despite the implementation of HIV/TB integration services at primary healthcare facility level, the effect of HIV on TB treatment outcomes has not been well investigated. To provide evidence base for TB treatment outcome improvement to meet End TB Strategy goal, we assessed the effect of HIV status on treatment outcomes of TB patients at a rural clinic in the Ugu Health District, South Africa.

Methods: We reviewed medical records involving a cohort of 508 TB patients registered for treatment between 1 January 2013 and 31 December 2015 at rural public sector clinic in KwaZulu-Natal province, South Africa. Data were extracted from National TB Programme clinic cards and the TB case registers routinely maintained at study sites. The effect of HIV status on TB treatment outcomes was determined by using multinomial logistic regression. Estimates used were relative risk ratio (RRR) at 95% confidence intervals (95%CI).

Results: A total of 506 patients were included in the analysis. Majority of the patients (88%) were new TB cases, 70% had pulmonary TB and 59% were co-infected with HIV. Most of HIV positive patients were on antiretroviral therapy (ART) (90% (n=268)). About 82% had successful treatment outcome (cured 39.1% (n=198) and completed treatment (42.9% (n=217)), 7% (n=39) died 0.6% (n=3) failed treatment, 3.9% (n=20) defaulted treatment and the rest (6.6% (n=33)) were transferred out of the facility. Using completed treatment as reference, HIV positive patients not on ART relative to

negative patients were more likely to have unsuccessful outcomes [RRR, 5.41; 95%CI, 2.11–13.86].

Conclusions: When compared HIV positive and HIV negative status, antiretroviral treatment had no effect on the likelihood of TB cure in rural primary care. The TB mortality rate in HIV positive patients, on the other hand, was higher than in HIV negative patients. Various HIV/TB indicators should be reviewed, and gaps filled to achieve the "End TB strategy" in rural South Africa.

Key words: TB; Treatment outcomes; co-infection; HIV Status, KwaZulu Natal province, South Africa.

1. Background

Tuberculosis (TB) is one of the major global infectious diseases with a high morbidity and mortality rate. Globally, an estimated 10.0 million (range 8.9–11.0 million) people fell ill with TB in 2019 [1]. There were 1.2 million (range, 1.1–1.3 million) TB deaths among human immunodeficiency virus (HIV) - negative people and an additional 208 000 (range, 177 000–242 000) TB deaths among HIV-positive people [1]. Ninety percent (90%) of all TB cases occur in adults, with a high prevalence in males compared to females [2]. The increase in incidence is also attributed to the development of multidrug resistant (MDR) and extremely drug resistant (XDR) strains of *Mycobacterium tuberculosis* [34]. Both MDR and XDR-TB are the causes of high TB mortality [4]. HIV prevalence in sub-Saharan Africa has significantly contributed to an increase in the incidence of TB [5]. An estimated 10 million people have been living with TB and 1.5 million have died in 2018 and the global burden of TB falls on 20 low- and middle-income (LMIC) countries, including sub-Saharan Africa [6]. The burden is attributed to the high rate of HIV/AIDS pandemics in those countries. Africa accounted for 84% of all TB/HIV deaths [6].

TB is the most common opportunistic infection and cause of death among PLWHIV in resource-limited countries [7, 8]. In the post-mortem period, the overall prevalence of TB was enormous and accounted for almost 40% of HIV-related facility-based deaths in resource-limited countries such as South Africa [9-11], Botswana [11, 12], Zimbabwe [11, 13, 14], Mozambique [11, 15], Uganda [11, 16] and Kenya [11, 17]. In contrast, the WHO reported 16% of HIV/TB related death in Africa [1].

South Africa (SA) ranked fifth among the highest TB incidences worldwide and first among TB/HIV co-infection cases with more than 65 percent of patients co-infected

with TB/HIV [54]. TB accounted for the third highest number of deaths in 20184 (6 %; n = 454,014), and combined TB and HIV accounted for 35.6% of all-cause mortality in SA [18-20]. The total number of people living with HIV in SA increased from an estimated 4.64 million in 2002 to 7.97 million by 2019 [21]. There are significant geographical variations in the rate of TB notification in SA which are not clearly correlated with the prevalence of HIV at the district level [21-23]. KwaZulu-Natal (KZN) carries the largest burden of HIV and related infections in SA, with HIV–TB co-infection estimated at approximately 70% [5, 24], and Ugu district reported 60.5% of TB/HIV co-infection rate [25].

According to recent data, 90% of people are aware of their HIV status of which 68% are on antiretroviral therapy (ART) and of which 87% are virally suppressed in SA [26]. ART may be associated with a reduced risk of HIV-associated TB disease in HIV-positive individuals due to a decrease in their viral load and an improvement in their immune system function [27]. On the one hand, while ART reduces new HIV infections, on the other hand, the marked decline in HIV-associated mortality has led to an increase in HIV prevalence and an increase in the number of life-years at TB risk. It is also plausible that HIV-positive people with TB infection may increase with CD4+ T-cell ART recovery, although the available data do not support this [27-29]. In addition, early initiation of ART during TB treatment (within 2–4 weeks) increased AIDS-free survival by 34–68% among patients with advanced HIV disease [11, 30-32]. Despite the inclusion of TB-HIV in the international and SA guidelines, the 2018 mortality rate for co-infected TB-HIV patients in SA was 73 (51-99) per 100,000 populations, which is more than three times higher than that for HIV-negative TB patients with a mortality rate of 37 (35–39) per 100,000 population [33]. TB incidence and mortality are declining in SA [7]. Data from a well-characterized rural SA

population with high HIV prevalence and TB incidence demonstrated considerable spatial heterogeneity in people with recently diagnosed TB, and has shown that every percentage increase in ART coverage was associated with a 2% decrease in the odds of recently diagnosed TB [23]

The End TB Strategy aims to reduce TB deaths by 90% and TB incidence rates by 80% in 2030 compared to 2015 [34]. Examining the various challenges that may impact on TB outcomes in rural primary health care in SA, the TB elimination target set for 2050 could be compromised if this dual burden of TB and HIV diseases is not controlled [5, 35]. The rate of TB incident stabilizes at a rate higher than that of the general population. These data highlight the need for more research into strategies for finding active cases in rural settings and the need to focus on strengthening primary health care [36]. Therefore, this retrospective study has been undertaken to evaluate TB outcomes in HIV positive patients in rural primary healthcare in Ugu Health District, KwaZulu-Natal, SA.

2. Methods

2.1. Study design

This retrospective cohort study of TB patients initiated on TB treatment was conducted from 1 January 2013 to 31 December 2015.

2.2 Study population

We included all patients diagnosed with TB irrespective of their age and HIV status in the study. Further, we included individuals that were registered as TB patients in 2012 and completed treatment or died in 2013. We also included as well as those that died or survived during the treatment period. Patients with unknown outcome or those with incomplete record were excluded. Basic demographic information including the age, gender, co-morbidities, tobacco Use, alcohol Use, substance use and duration on treatment were collected.

2.3 Study setting

The Ugu Health District is in the rural aspects of the KwaZulu-Natal (KZN) province (Figure 1). Ugu district has a population of 733 228 people [37]. During the study period, Ugu district had the highest HIV prevalence and TB incidence of any district in KZN, 41.7% and 1096 per 100,000 people, respectively [37]. In terms of infectious TB (pulmonary smear-positive), Ugu ranks 12th, with 325 cases per 100,000 people, which is higher than the country's average of 208 cases per 100,000 people [37]. Elim clinic, a primary health care facility was selected based on convenience, the study goal and the availability of information on HIV and TB infections".

2.4. Sample size

A total of 478 cases were estimated to be adequate sample that would have the power to detect a significant difference in mortality between HIV positive and negative TB patients on anti-tuberculosis treatment. We used a 95% precision of estimate with power of 80% and estimated risk difference of 6% in outcome between exposed and unexposed.

2.5. Participant selection and definitions

All TB patients of all ages recorded in the TB treatment register, with HIV positive status and started on treatment were included in this study. These were all TB patients on treatment attending care at Elim Clinic, in Ugu District, KZN province within the stipulated time. At admission, patients were screened for other comorbidities including HIV infection. After following the treatment, the outcomes of treatment were recorded in seven mutually exclusive categories. Patients were classified as cured, completed treatment, interrupted treatment, moved, transferred out, failed and died. The main interest of this study was to compare the likelihood of observing two main outcomes. These included being cured versus all other outcomes including death and the likelihood of dying versus staying alive regardless of the outcome of treatment. The World Health Organization (WHO) defines “cured” as follows: a pulmonary TB patient with bacteriologically confirmed TB at the start of treatment who was smear or culture negative in the last month of treatment and on at least one previous occasion, and "treatment completed": A TB patient who completed treatment without evidence of failure but with no record to show that sputum smear or culture results in the last month of treatment and on at least one previous occasion were negative either because tests were not done or because results are unavailable [38].

2.6 Outcome and exposures

The first outcome of interest was "cured" defined as 1 if the patient was declared "cured" by the healthcare team or 0 if otherwise. The second outcome was "death" coded 1 if the patient was declared dead and 0 if otherwise. Given the small number of deaths which were 35 in total, we created a nominal outcome, which captured all outcomes and combined them into 3 categories: cured, dead and other outcomes.

2.7. Data collection

Data were extracted from the TB register using structured data collection form from all the TB patients initiated on treatment at this rural public health clinic from 1 January 2013 and 31 December 2015. The data consists of collecting socio-demographic factors (age and gender), TB related factors (categories, pre-treatment smear, type, site, outcome duration, regimen at the baseline) and other factors such as ART initiation and co-trimoxazole prophylaxis, comorbidities, tobacco, and alcohol use. A data dictionary was formulated to guide data cleaning and analysis. No personal identifiers were extracted from the manual medical records.

2.8. Data management

Data were entered on excel spread sheet and imported into Stata software. This step was followed by thorough checking to assess missing values. Data were coded into numerical values and continuous data categorised; clean data were imported into Stata version 15 for analysis.

2.9. Data Analysis

The main interest of this study was to compare the likelihood of observing two main outcomes. These included being cured versus all other outcomes including death and the likelihood of dying versus staying alive regardless of the outcome of treatment. Data were summarized and presented in tables. As part of the descriptive statistics for

the population under study, summary tables were constructed to describe variables in terms of their frequency distribution. Pearson Chi-square and Fisher Exact tests were used to test association between independent variables and TB outcomes (cured, completed treatment, interrupted treatment, moved, transferred out, failed, and died). Given the fact that cured was a common outcome (39%), we fitted a log-binomial regression model to estimate the relative risk of being declared cured versus not being cured (Table 3). Finally, we fitted a multinomial logistic regression model predicting the likelihood of cured and death versus other outcomes. This allowed us to make some predictions on death, as a function of the selected socio-demographic covariates. Odds ratios were computed to assess the association between HIV status and TB mortality. Precision of estimate of the odds ratio was set at 5% significance level. All factors with $p \leq 0.1$ on univariate analysis were further analysed in multiple logistic regression model.

2.5. Ethical considerations

Ethical approval was granted by the Monash University Human Research Ethics Committee and permission to conduct the study and access the medical records at Elim Primary Health Care Clinic was obtained from the District Manager of Ugu Health District, KwaZulu Natal province. Waiver of consent was applied for and obtained since the data were retrieved from older clinic records. Data entry and analysis were done anonymously, and no attempt was made to link data to the individual identifier.

3. Results

Overall, the study included 508 patients. Of this total, 2 patients were dropped from further analysis because one was transferred to another facility the same day of admission to the hospital and the other one was on prophylaxis TMP-SMX and was

HIV negative. Hence, our final analytical sample included 506 patients for whom we had valid data on the TB treatment outcomes.

Table 1 reported the demographic characteristics and TB outcomes. The findings revealed that 39.13% (n=198) were reported as being cured at the end of treatment. The remaining 60.87% patients were classified as dead or alive, but not cured. Some patients were transferred to other facilities, whereas other were classified as treatment interrupted, transferred to another district, treatment outcome unknown, treatment completed and moved to another facility in the same district. Table 1 shows significant differences with regards to TB cured in terms of age, tobacco, and alcohol uses. For instance, younger patients had lower proportion of TB cure -(22.62% of those less than 20 years of age) compared to older patients. Among tobacco users, we found that about 60% were TB cured, whereas among non-tobacco users only 37% were cured. Likewise, the findings estimated that alcohol users, had higher proportion of being TB cured (57.14% of all alcohol users) while non-alcohol users' patients only (38.08%) were cured.

The overall TB related mortality rate was 6.92% (n=35 patients). The mortality rate was significantly associated with HIV status. For instance, the mortality rate was higher among HIV positive patients (9.67%, i.e. 29 patients out of 300 patients) compared to HIV negative patients among whom only for 2.91% was reported (6 patients out of 206 HIV negative patients). Given the small sample size for the mortality rate, this was impractical to perform further analysis and obtain reliable estimates.

Table 1. Characteristics of TB patients presenting to Elim Clinic on the basis of TB cured outcomes

Factors	Not Cured n (%)	Cured n (%)	P-value	Alive) n (%)	Dead n (%)	p-value
	**308 (60.87)**	**198 (39.13)**		**471 (93.08)**	**35 (6.92)**	
**Duration on treatment[1]**	5.55 (0.16)	6.15 (0.13)	0.009*	6.01 (0.11)	2.74 (0.44)	0.001*
**HIV Status**						
Negative	123 (59.71)	83 (40.29)	0.658	200 (97.09)	6 (2.91)	0.004*
Positive	185 (61.67)	115 (38.33)		271 (90.33)	29 (9.67)	
**Age-group**						
<20	65 (77.38)	19 (22.62)	0.018*	82 (97.62)	2 (2.38)	0.101[F]
20 – 29	78 (58.21)	56 (41.79)		124 (92.54)	10 (7.46)	
30 – 39	72 (55.38)	58 (44.62)		120 (92.31)	10 (7.69)	
40 – 49	46 (58.97)	32 (41.03)		75 (96.15)	3 (3.85)	
50+	47 (58.75)	33 (41.25)		70 (87.50)	10 (12.50)	
**Sex**						
Female	138 (61.61)	86 (38.39)	0.762	205 (91.52)	19 (8.48)	0.216
Male	170 (60.28)	112 (39.72)		266 (94.33)	16 (5.67)	
**Comorbidity**						
No	286 (60.98)	183 (39.02)	0.855	438 (93.39)	31 (6.61)	0.311[F]
Yes	22 (59.46)	15 (40.54)		33 (89.19)	4 (10.81)	
**Tobacco Use**						
No	292 (62.66)	174 (37.34)	0.005*	432 (92.70)	34 (7.30)	0.345[F]
Yes	16 (40.00)	24 (60.00)		39 (97.50)	1 (2.50)	
**Alcohol Use**						
No	296 (61.92)	182 (38.08)	0.045*	443 (92.68)	35 (7.32)	0.246[F]
Yes	12 (42.86)	16 (57.14)		28 (100.00)	0 (0.00)	
**Substance Use**						
No	292 (60.08)	194 (39.92)	0.101	452 (93.00)	34 (7.00)	1.00[F]
Yes	16 (80.00)	4 (20.00)		19 (95.00)	1 (5.00)	

Note: [1] Duration of treatment in months

All p-value from Pearson Chi-square test, unless otherwise determined.

** p-value < 0.05; [F] indicates results from Fisher Exact test*

Table 2: Characteristics of TB patients presenting to Elim Clinic on the basis of HIV status.

Characteristics	HIV Negative n(%) 178 (37.2)	HIV Positive n(%) 300 (62.8)	P-value
**Categories of TB**			
New	161 (91.0)	259 (86.3)	0.395¶
RAC	8 (4.5)	25 (8.3)	
RF	5 (2.8)	8 (2.7)	
RI	3 (1.7)	8 (2.7)	
**Pre-treatment sputum smear status**			
Negative	27 (29.7)	41 (30.4)	0.910‡
Positive	64 (70.3)	94 (69.6)	
**Type of Tuberculosis**			
Pulmonary	124 (69.7)	210 (70.0)	0.938‡
Extra-Pulmonary	54 (30.3)	90 (30.0)	
**Site of Tuberculosis**			0.047*¶
Bones/Joints	1 (2.0)	0 (0.0)	
Lymph Nodes	0 (0.0)	4 (4.5)	
Miliary	4 (7.8)	6 (6.7)	
Meningitis	2 (3.9)	17 (19.1)	
Primary	12 (23.5)	17 (19.1)	
Pleura	12 (23.5)	22 (24.7)	
Other organs	20 (39.2)	23 (25.8)	
**Receiving ART**			
No	172 (100.0)	30 (10.1)	<0.001*¶
Yes	0 (0.0)	267 (89.9)	
**Co-trimoxazole Prophylaxis**			
No	173 (100.0)	22 (7.3)	<0.001*¶
Yes	0 (0.0)	278 (92.7)	
**Outcome duration(days)**	184 (176-203)**	184 (173-209)*	0.7547†
**Comorbidity**			
No	155 (87.1)	286 (95.3)	0.001*
Yes	23 (12.9)	14 (4.7)	
**Regimen type at baseline**			
HRZE	157 (88.7)	280 (93.4)	0.005*¶
HR	15 (8.5)	10 (3.3)	
RHZ	5 (2.8)	3 (1.0)	
HRZES	0 (0.0)	7 (2.3)	

N- Total number in each group; % - row percentages; Numbers may not add-up because of missing variables.

*Significant p-value;

**Median and Inter-quartile ranges (IQR); Test statistic based on ¶Fisher's Exact, ‡Chi-square and Wilcoxson †Rank-sum test.

HIV – Human Immunodeficiency Virus; ART – Antiretroviral therapy; Categories of TB treatment: RAC – retreatment after completion of a previous course without microscopic result; RF – Treatment failure: a patient who, while on treatment, remained or became again smear positive five months or later after commencing treatment; RI – Treatment after interruption: a patient whose treatment is interrupted for two or more months and who returned to the health service.

HRZE – Isoniazid, Rifampicin, Pyrazinamide and Ethambutol for New adult patients; HRZ – Isoniazid, Rifampicin and Pyrazinamide for New Paediatric patients; HRZES – Isoniazid, Rifampicin, Pyrazinamide, Ethambutol and Streptomycin for Retreatment patients.

Table 3 below presented the results from the univariate and multivariate log-binomial model, which sought to identify covariates associated with cure event. These factors included 'duration of treatment, age, comorbidity, tobacco, alcohol use, and HIV positive status' which was our main exposure of interest

We found that HIV positive status showed to affect the likelihood of TB cure among patients on TB treatment. The results seem to suggest that HIV positive patients were 12% less likely to be cured from TB compared to the HIV negative patients, however this finding was not statistically significant (RR: 0.88; 95% C.I.: 0.69 – 1.11). The length of TB treatment was associated with TB cure. The results show that for each additional month on TB treatment there was a 3% significantly increased likelihood of TB cure (RR: 1.03; 95% C.I.: 1.01 – 1.06). Alcohol use and Tobacco smoking were not significantly associated with increased risk of cure.

Table 3. Factors associated with **cure** among TB patients presenting for care at Elim Clinic

Factors	Univariate Analysis RR (95% C.I.)	Adjusted Analysis RR (95% C.I.)	p-value
**Duration on treatment**	**1.02 (1.01 – 1.05)**	**1.03(1.01 – 1.06)**	0.039
**HIV Status**			
Negative	Ref	Ref	
Positive	0.95 (0.76-1.19)	0.88(0.69-1.11)	0.124
**Age-group**			
<20	Ref	Ref	
20 – 29	1.85(1.19 – 2.88)	1.82(1.15 – 2.91)	0.014
30 – 39	1.97(1.27 -3.06)	2.00(1.26 – 3.17)	0.008
40 – 49	1.81(1.13 – 2.92)	1.79(1.10 – 2.91)	0.033
50+	1.82(1.13 – 2.93)	1.77(1.09 – 2.85)	0.036
**Sex**			
Female	Ref	-	
Male	1.03(0.83 – 1.29)	-	
**Comorbidity**			
No	Ref	-	
Yes	1.04(0.69 – 1.56)	-	
**Tobacco Use**			
No	Ref	Ref	
Yes	1.60(1.22 – 2.12)	1.54(0.95 – 2.48)	0.117
**Alcohol Use**			
No	Ref	Ref	
Yes	1.5(1.07 – 2.11)	0.93(0.51 – 1.69)	0.909

Table 4 below present's results from the multinomial logistic regression model with two levels of outcomes 'dead and other'. We found that HIV status and age were associated with increased mortality among TB patients on treatment. The results shows that HIV positive TB patients were nearly 4 times likely to die of TB compared to the HIV negative patients, and this finding was statistically significant (RRR: 3.73; 95% C.I.: 1.24 – 11.19). Furthermore, older age was associated with 5 times increased risk of dying among TB patients aged 50 years and above, however this was not statistically significant (RR: 4.99; 95% C.I. 0.88-28.50). In addition, duration of TB

treatment was associated with reduced risk of dying. We found that for each additional month on TB treatment there was a 44% significant reduction in the risk of TB mortality (RR: 0.56; 95% C.I.: 0.47 – 0.66).

Table 4. Risk factors associated with death among TB patients presenting for care at Elim Clinic

Factors	Univariate Analysis RRR (95% C.I.)	Adjusted Analysis RRR (95% C.I.)
**Duration on treatment**	0.54 (0.46 – 1.13)	0.56 (0.47 – 0.66)
**HIV Status**		
Negative	Ref	Ref
Positive	3.63 (1.46 – 9.02)	3.73 (1.24 – 11.19)
**Age group**		
<20	Ref	Ref
20 – 29	4.63 (0.98 – 21.97)	2.40 (0.45 – 12.78)
30 – 39	5.08 (1.07 – 24.13)	2.18 (0.39 – 12.29)
40 – 49	2.20 (0.35 – 13.71)	1.46 (0.20 – 10.83)
50+	8.51 (1.77 – 40.98)	4.99 (0.88 – 28.50)
**Sex**		
Female	Ref	Ref
Male	0.65 (0.32 – 1.32)	0.61 (0.26 – 1.46)
**Co-morbidity**		
No	Ref	Ref
Yes	1.83 (0.58 – 5.75)	1.30 (0.29 – 5.80)
**Tobacco Use**		
No	Ref	Ref
Yes	0.51 (0.06 – 3.95)	0.56 (0.06 – 4.96)

*****_other outcomes include…. Alive, transferred, defaulted etc_

4. Discussion

This study evaluated the effect of HIV status and antiretroviral treatment on the treatment outcome of TB patients in a primary healthcare facility in rural South Africa. The study comprised of 282 males (55.7) and 224 females (44.3) with a median age of 32 (IQR: 23-43) years. The main outcomes of the study were categorized as cured or dead or other. This study found that 39.13% (198/506) of the patients were cured of Tuberculosis, of which 115 were HIV positive. Even though the patient's records did not clarify that TB patients completed a full course of treatment, our findings shows that the treatment success rate may be estimated as below the WHO target of 90% [39]. The overall TB related mortality rate was 6.92% (n=35 patients) and the study showed that the risk of mortality rate was significantly associated with HIV status. For instance, the mortality rate was higher among HIV positive patients (9.67%, i.e., 29 patients out of 300 patients) compared to HIV negative patients (2.91%, 6 patients out of 206 HIV negative patients). Longer treatment period was associated with a lowered risk of death; however, HIV positive status of a TB patient had no effect on the likelihood of TB cure when compared to HIV negative status of a TB patient.

In reviewing other studies conducted in rural Africa, HIV status did not appear to have any effect on successful treatment of TB, and this could be due to lack of sufficient data in a study conducted in Ghana [40], however, the same study showed that HIV status was associated with death [40]. Our study found a close association between HIV-positive status and increased risk of dying of TB like the Ghana study that showed a higher mortality rate among HIV/TB co-infected individuals [40]. Another study in Tanzania found an EPTB prevalence of 5.6% with a high mortality rate of 14.3% and a combined death/LTFU rate of 46.8% among people living with HIV [41]. Patients with EPTB not receiving ART and >45 years of age were at a higher risk for poor outcomes

[41]. There were no differences in treatment success rates for HIV-positive TB patients on ART and HIV-negative in Kenya [42]. The similarities and differences between studies could be explained by the levels of ART uptake and TB diagnosis. Following a review several of these studies, our study finding is consistent with the study conducted in Kenya, owing to the ongoing increasing scale-up and uptake of ART among HIV positive individuals in both South Africa and Kenya. In Table 2 we show an ART uptake rate of 89.9%, compared to 61% in Kenya [42]. In contrast, 3.7% of ART uptake was recorded in Ghana [40] and 16% patients with extrapulmonary TB did not receive ART in Tanzania [41]. Aside from that, our study found 70.0% of patients to have pulmonary TB, compared to 36.9% in a Tanzanian study [41]. In fact, pulmonary TB is easier to diagnose than other types of TB. Previous data shows that Extrapulmonary TB is associated with poor TB outcome in HIV-infected people [43]. In our study low number of people were on ART at that time as the scaling up program was still in its infancy stage, hence difficult to make a meaningful analysis to assess its impact on TB outcome.

Age was reported as significantly associated with being cured. In our study, older patients were less likely to be cured and more likely to die of TB compared to younger patients-. These results are in line with the study conducted in Zimbabwe where elderly patients had a higher risk of not being cured and high risk of dying [44]. Another study reported that older age patients are more likely to develop extra-pulmonary and atypical forms of TB disease that are often harder to diagnose than sputum smear-positive pulmonary tuberculosis [45]. Extra pulmonary and smear negative PTB have all previously shown to be associated with high TB mortality [43, 46, 47].

Existing evidence from previous meta-analysis of high-quality studies has shown that smoking tobacco was noted to influence the outcome of TB treatment as smokers had a likelihood of being cured compared with non-smokers (OR of 2.6 (95%CI 2.1–3.4) [48]. Further, the associations between smoking and TB mortality showed a pooled OR of 1.3 (95%CI 1.1–1.6) [48]. Another large systematic review showed that cigarette smoking was significantly linked with poor TB treatment outcomes [49]. This shows that evidence on the effect of smoking on TB outcomes remains inconclusive. The findings of our study however confirms what has been previously reported showing an association between smoking tobacco and TB cure. Smoking tobacco affects both innate and adaptive immunity, weakening the immunological defensive system in humans [50]. This appears to be the reason why smokers are at a higher risk of developing extra-pulmonary tuberculosis [51]. Apart from this, smoking tobacco increases the risk of mycobacterium TB infection as well as the development of tuberculosis in infected individuals [52]. However, it does not have a negative effect on cure among those on TB treatment.

A recent study in the KZN revealed that staff rotation challenges across different services have been contributed to skills insufficiency (80%); staff shortage (50%); high staff turnover, absenteeism, and staff personal preferences (30%) in the KZN [5]. Despite a substantial investment in the clinical expansion program in SA, sparsely populated rural areas are still constrained in geographic distribution and access to health services (seen at a lower PHC utilization rate) and this is a major challenge in provision HIV and TB services in rural areas [33]. In this context, integrated services could enhance and promote equity as they maximize the potential benefit for each facility visit for health care access [33].

Besides this, a large TB cohort in Western Cape, South Africa showed that concomitant diseases were not associated with TB mortality [53]. This has been clearly shown in our study. However, in the same study, factors such as 'treatment category' and 'comorbidity' were significantly associated with unsuccessful TB treatment [53].

The strength of our study is our sample size and its representativeness of the rural population, thereby minimizing selection bias. It was sufficient to estimate HIV/TB outcomes in rural primary care settings. Our study has several limitations that could lead to underestimation of HIV/TB outcomes. Retrospective cohort design uses records that have already been collected. This is an inherent weakness of the nature of use of existing medical records as data used is dependent on data documented in the records. We did not obtain the information on treatment completed outcome, HIV viral load and CD4 cells as this information was not available in the patients' medical records. As such, we were unable to accurately link all our patients' outcomes to these specific exposures. It is also possible that some of the patients included in our study were misclassified based on exposure or outcome status during analysis.

5. Conclusion

In conclusion, HIV positive status, and length of treatment were significantly associated with TB treatment outcomes in this rural primary care facility. The risk of dying was higher among the HIV positive TB patients while duration on treatment was associated with increased likelihood of cure and reduced risk of mortality. Despite a higher ART coverage in general, HIV status was a strong predictor of the TB mortality in rural SA. However, the TB treatment success rate in rural SA was lower than the WHO target. This study could have a significant impact on the reinforcement of HIV/TB program integration in rural SA. To achieve the End TB strategy in rural SA, HIV/TB integration services needs to be reviewed to identify potential challenges in its implementation. Furthermore, various HIV/TB indicators should be reviewed to allow for strengthening of TB/HIV integration monitoring approach by the TB program.

6. Supporting information

S1 Data analysis commands

7. Acknowledgements: The authors would like to thank Ugu Health District management for the permission to conduct the study and the staff of Elim Clinic for the support provided during the duration of this study.

~~7.~~8. Author Contributions

Conceptualization: Peter S Nyasulu, Jabulani Ncayiyana

Data curation: Peter S Nyasulu, Lovemore N Sigwadhi, Emery Ngasama, Teye Umanah, Jabulani Ncayiyana

Formal analysis: Lovemore N Sigwadhi, Emery Ngasama, Lovelyn U Ozougwu, Ruvimbo BC Nhandara, Teye Umanah, Birhanu T Ayele

Investigation: Peter S Nyasulu, Emery Ngasama, Jacques L Tamuzi, Birhanu T Ayele, Jabulani Ncayiyana.

Methodology: Peter S Nyasulu, Emery Ngasama, Jacques L Tamuzi, Lovemore N Sigwadi, Birhanu T Ayele, Jabulani Ncayiyana.

Supervision: Peter S Nyasulu, Birhanu T Ayele, Jabulani Ncayiyana

Writing – original draft: Peter S Nyasulu, Jacques L Tamuzi, Lovelyn U Ozougwu Ruvimbo BC Nhandara

Writing – review & editing: Peter S Nyasulu, Emery Ngasama, Jacques L Tamuzi, Lovemore N Sigwadhi, Lovelyn U Ozougwu, Ruvimbo BC Nhandara, Birhanu T Ayele, Teye Umanah, Jabulani Ncayiyana.

Financial Disclosure: The author(s) received no specific funding for this work.

Competing Interests: The authors have declared that no competing interests exist.

Ethics Statement: **Ethical approval to conduct the study was obtained from the Monash University Research Ethics Committee.**

Data Availability: Dataset are available upon request by submitting the request form to the corresponding author (email: pnyasulu@sun.ac.za).

~~8.~~9. References

1. World Health Organization. Global tuberculosis report, 2020. Available from: https://apps.who.int/iris/bitstream/handle/10665/336069/9789240013131-eng.pdf.
2. Department of Health (Republic of South Africa). National Tuberculosis Management Guidelines, 2009. Available from: https://www.tbonline.info/media/uploads/documents/south_african_national_tuberculosis_management_guidelines_%282009%29.pdf
3. Roy E, Lowrie DB, Jolles SR. Current strategies in TB immunotherapy. Curr Mol Med. 2007;7(4):373-86.
4. Alemu A, Bitew ZW, Worku T, Gamtesa DF, Alebel A. Predictors of mortality in patients with drug-resistant tuberculosis: A systematic review and meta-analysis. PLoS One. 2021;16(6): e0253848.
5. Kalonji D, Mahomed OH. Health system challenges affecting HIV and tuberculosis integration at primary healthcare clinics in Durban, South Africa. Afr J Prim Health Care Fam Med. 2019;11(1): e1-e7.
6. World Health Organization. HIV-Associated Tuberculosis, 2019. Available from: https://www.who.int/tb/areas-of-work/tb-hiv/tbhiv_factsheet.pdf?ua=1.
7. Naidoo K, Gengiah S, Yende-Zuma N, Padayatchi N, Barker P, Nunn A, Subrayen P, Abdool Karim SS. Addressing challenges in scaling up TB and HIV treatment integration in rural primary healthcare clinics in South Africa (SUTHI): a cluster randomized controlled trial protocol. Implement Sci. 2017;12(1):129.
8. Manosuthi W, Wiboonchutikul S, Sungkanuparph S. Integrated therapy for HIV and tuberculosis. AIDS Res Ther 2016; 13(1):1-12.

9. Rennert WP, Kilner D, Hale M, Stevens G, Stevens W, Crewe-Brown H. Tuberculosis in children dying with HIV-related lung disease: clinical-pathological correlations. Int J Tuberc Lung Dis 2002; 6:806–813.
10. Wong EB, Omar T, Setlhako GJ, Osih R, Feldman C, Murdoch DM, et al. Causes of death on antiretroviral therapy: a post-mortem study from South Africa. PLoS One 2012; 7: e47542.
11. Gupta Rishi K, Lucas Sebastian B, Fielding Katherine L, Lawn Stephen D. Prevalence of tuberculosis in post-mortem studies of HIV-infected adults and children in resource-limited settings: a systematic review and meta-analysis. AIDS (London, England) 2015; 29(15):1987-2002.
12. Ansari NA, Kombe AH, Kenyon TA, Hone NM, Tappero JW, Nyirenda ST, et al. Pathology and causes of death in a group of 128 predominantly HIV-positive patients in Botswana, 1997-1998. Int J Tuberc Lung Dis 2002; 6:55–63.
13. Ikeogu MO, Wolf B, Mathe S. Pulmonary manifestations in HIV seropositivity and malnutrition in Zimbabwe. Arch Dis Child 1997; 76:124–128.
14. Nathoo KJ, Gondo M, Gwanzura L, Mhlanga BR, Mavetera T, Mason PR, et al. Fatal Pneumocystis carinii pneumonia in HIV-seropositive infants in Harare, Zimbabwe. Trans R Soc Trop Med Hyg 2001; 95:37–39.
15. Carrilho C, Monteiro E, Ussene E, Macie A, Fernandes F, Lorenzoni C, et al. Causes of death in HIV/AIDS patients in Maputo Central Hospital: a retrospective study from 2011. Histopathology 2012; 61:1–2
16. Cox JA, Lukande RL, Nelson AM, Mayanja-Kizza H, Colebunders R, Van Marck E, et al. An autopsy study describing causes of death and comparing

clinico-pathological findings among hospitalized patients in Kampala. Uganda PLoS One 2012; 7: e33685.

17. Chakraborty R, Pulver A, Pulver LS, Musoke R, Palakudy T, D'Agostino A, et al. The postmortem pathology of HIV-1-infected African children. Ann Trop Paediatr 2002; 22:125–131.

18. Stats sa. TB tops leading causes of death in SA in 2018. Available at http://www.statssa.gov.za/?p=14435.

19. Pillay-van Wyk V, Bradshaw D. Mortality, and socioeconomic status: the vicious cycle between poverty and ill health. Lancet Glob Health. 2017;5(9): e851-e852.

20. Statistic South Africa. Mid-year population estimates, 2019. Available from: https://www.statssa.gov.za/publications/P0302/P03022019.pdf.

21. Nanoo A, Izu A, Ismail NA, Ihekweazu C, Abubakar I, Mametja D, Madhi SA. Nationwide and regional incidence of microbiologically confirmed pulmonary tuberculosis in South Africa, 2004-12: a time series analysis. Lancet Infect Dis. 2015;15(9):1066-1076.

22. Sloot R, Maarman GJ, Osman M, Marx FM. On Behalf of The Desmond Tutu TB Centre-Working Group Data Analysis and Modelling FM. Variation in HIV prevalence and the population-level effects of antiretroviral therapy in reducing tuberculosis incidence in South Africa. S Afr Med J.;108(8):12370.

23. Tomita A, Smith CM, Lessells RJ, Pym A, Grant AD, de Oliveira T, Tanser F. Space-time clustering of recently diagnosed tuberculosis and impact of ART scale-up: Evidence from an HIV hyper-endemic rural South African population. Sci Rep. 2019;9(1):10724.

24. Health System Trust. Durban. District Health Barometer 2015/16. Available at https://www.hst.org.za/publications/District%20Health%20Barometers/District%20Health%20Barometer%202015_16.pdf

25. Health Systems Trust. Tuberculosis, 2015. Available at https://www.hst.org.za/publications/District%20Health%20Barometers/9%20(Section%20A)%20Tuberculosis.pdf

26. UNAIDS. UNAIDS data 2019. Available at https://www.unaids.org/sites/default/files/media_asset/2019-UNAIDS-data_en.pdf

27. van Halsema CL, Fielding KL, Chihota VN, George EC, Lewis JJ, Churchyard GJ, Grant AD. Brief Report: The Effect of Antiretroviral Therapy and CD4 Count on Markers of Infectiousness in HIV-Associated Tuberculosis. J Acquir Immune Defic Syndr. 2015;70(1):104-8.

28. Khan PY, Crampin AC, Mzembe T, Koole O, Fielding KL, Kranzer K, Glynn JR. Does antiretroviral treatment increase the infectiousness of smear-positive pulmonary tuberculosis? Int J Tuberc Lung Dis. 2017;21(11):1147-1154.

29. Abdool Karim SS, Naidoo K, Grobler A, Padayatchi N, Baxter C, Gray AL, Gengiah T, Gengiah S, Naidoo A, Jithoo N, Nair G, El-Sadr WM, Friedland G, Abdool Karim Q. Integration of antiretroviral therapy with tuberculosis treatment. N Engl J Med. 2011;365(16):1492-501.

30. Blanc FX, Sok T, Laureillard D, Borand L, Rekacewicz C, Nerrienet E, et al. Earlier versus later start of antiretroviral therapy in HIV-infected adults with tuberculosis. New England Journal of Medicine 2011; 365(16):1471-81.

31. Havlir DV, Kendall MA, Ive P, Kumwenda J, Swindells S, Qasba SS, et al. Timing of antiretroviral therapy for HIV-1 infection and tuberculosis. New England Journal of Medicine 2011; 365(16):1482-91.

32. World Health Organization. WHO Global Tuberculosis Report -— South Africa profile, 2019. Available from: file:///D:/Files/The%20burden%20of%20TB-COVID-19%20in%20Sub-Saharan%20Africa/2019%20WHO%20Global%20Tuberculosis%20Report%20-%20South%20Africa%20profile.pdf

33. Scott V, Sanders D. Evaluation of how integrated HIV and TB programs are implemented in South Africa and the implications for rural-urban equity 2013. Available from: http://repository.uwc.ac.za/handle/10566/1111

34. World Health Organization. Global tuberculosis report (executive summary), 2018. Available from: https://www.who.int/tb/publications/global_report/tb18_ExecSum_web_4Oct18.

35. Kwan CK, Ernst JD. HIV and tuberculosis: a deadly human syndemic. Clin MicrobiolRev 2011; 24(2):351-76.

36. Houlihan CF, Mutevedzi PC, Lessells RJ, Cooke GS, Tanser FC, Newell ML. The tuberculosis challenge in a rural South African HIV programme. BMC Infect Dis. 2010; 10:23.

37. Department of Health Province/KwaZulu Natal. District Health Plan 2015/2016. UGU KwaZulu-Natal, 2015. Available at http://www.kznhealth.gov.za/Strategic/DHP/2015-16/ethekwini.pdf

38. World Health Organization. Treatment of tuberculosis: guidelines, 2010. Available at

https://apps.who.int/iris/bitstream/handle/10665/44165/9789241547833_eng.pdf

39. World Health Organization. Global Tuberculosis 2018 report, 2018. Available at https://www.who.int/tb/publications/global_report/gtbr2018_main_text_28Feb2019.pdf

40. Ogyiri L, Lartey M, Ojewale O, Adjei AA, Kwara A, Adanu RM, Torpey K. Effect of HIV infection on TB treatment outcomes and time to mortality in two urban hospitals in Ghana-a retrospective cohort study. Pan Afr Med J. 2019; 32:206.

41. Arpagaus A, Franzeck FC, Sikalengo G, Ndege R, Mnzava D, Rohacek M, Hella J, Reither K, Battegay M, Glass TR, Paris DH, Bani F, Rajab ON, Weisser M; KIULARCO Study Group. Extrapulmonary tuberculosis in HIV-infected patients in rural Tanzania: The prospective Kilombero and Ulanga antiretroviral cohort. PloS One. 2020;15(3):e): e0229875.

42. Owiti P, Zachariah R, Bissell K, Kumar AM, Diero L, Carter EJ, Gardner A. Integrating tuberculosis and HIV services in rural Kenya: uptake and outcomes. Public Health Action. 2015;5(1):36-44.

43. Mabunda TE, Ramalivhana NJ, Dambisya YM. Mortality associated with tuberculosis/HIV co-infection among patients on TB treatment in the Limpopo province, South Africa. Afr Health Sci. 2014;14(4):849-54.

44. Ncube RT, Takarinda KC, Zishiri C, Van den Boogaard W, Mlilo N, Chiteve C, et al. Age-stratified tuberculosis treatment outcomes in Zimbabwe: are we paying attention to the most vulnerable? Public health action 2017;7(3):212-7.

45. Negin J, Abimbola S, Marais BJ. Tuberculosis among older adults-—time to take notice. Int J Infect Dis. 2015; 32:135-7.

46. Rao VK, Iademarco EP, Fraser VJ, Kollef MH. The impact of comorbidity on mortality following in-hospital diagnosis of tuberculosis. Chest. 1998; 114:1244–1252.

47. Peter JG, Theron G, Singh N, Singh A, Dheda K. Sputum induction to aid diagnosis of smear-negative or sputum-scarce tuberculosis in adults in HIV-endemic settings. Eur Respir J. 2014;43(1):185-94.

48. Slama K, Chiang CY, Enarson DA, Hassmiller K, Fanning A, Gupta P, Ray C. Tobacco, and tuberculosis: a qualitative systematic review and meta-analysis. Int J Tuberc Lung Dis. 2007;11(10):1049-61.

49. Burusie A, Enquesilassie F, Addissie A, Dessalegn B, Lamaro T. Effect of smoking on tuberculosis treatment outcomes: A systematic review and meta-analysis. PLoS One. 2020;15(9): e0239333.

50. Qiu F, Liang C-L, Liu H, Zeng Y-Q, Hou S, Huang S, Lai X, et al. Impacts of cigarette smoking on immune responsiveness: Up and down or upside down? Oncotarget 2017, 8(1):268.

51. Maurya V, Vijayan VK, Shah A. Smoking and tuberculosis an association overlooked. The international journal of tuberculosis and lung disease: the official journal of the International Union against Tuberculosis and Lung Disease 2002, 6(11):942–951.

52. Bates MN, Khalakdina A, Pai M, Chang L, Lessa F, Smith KR. Risk of tuberculosis from exposure to tobacco smoke: a systematic review and meta-analysis. Archives of internal medicine 2007, 167(4):335–342.

53. Azeez A, Ndege J and Mutambayi R. Associated factors with unsuccessful tuberculosis treatment outcomes among tuberculosis/HIV coinfected patients

with drug-resistant tuberculosis. International Journal of Mycobacteriology 2018; 7(4): 347.

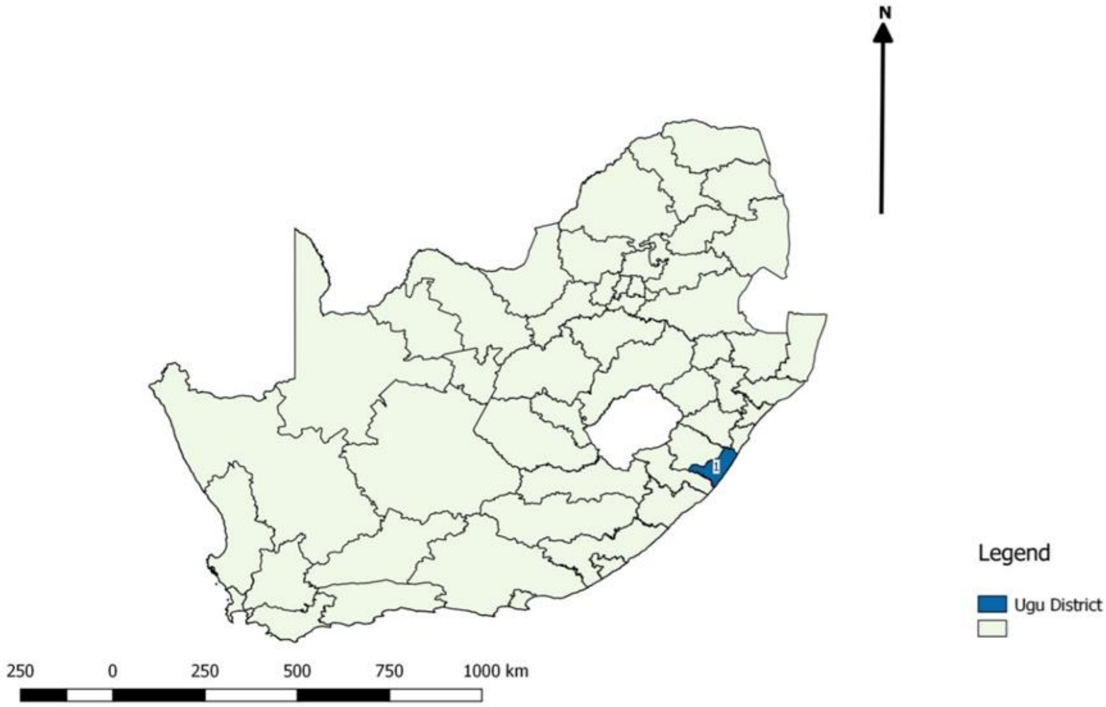

Figure 1: Map of study setting: Ugu District in KwaZulu Natal, South Africa

Reviewers’ responses to reviewers

Title: Effect of HIV status and antiretroviral treatment on treatment outcomes of tuberculosis patients in a rural primary healthcare clinic in South Africa

Journal Requirements:

Comment 1. Please ensure that your manuscript meets PLOS ONE's style requirements, including those for file naming. The PLOS ONE style templates can be found at https://journals.plos.org/plosone/s/file?id=wjVg/PLOSOne_formatting_sample_main_body.pdf and https://journals.plos.org/plosone/s/file?id=ba62/PLOSOne_formatting_sample_title_authors_affiliations.pdf

Response: *Thanks, we have revised the manuscript in accordance with the PLOS One formatting sample.*

Comment 2. In the ethics statement in the manuscript and in the online submission form, please provide additional information about the patient records used in your retrospective study, including a) whether all data were fully anonymized before you accessed them; b) the date range (month and year) during which patients' medical records were accessed; c) the date range (month and year) during which patients whose medical records were selected for this study sought treatment. If the ethics committee waived the need for informed consent, or patients provided informed written consent to have data from their medical records used in research, please include this information.

Response: Thanks for the comments and suggestions. Data collection for this study commenced from the 1st of September 2016 and ended on 31st of October 2016. The data clerk was contracted for a 2-month period to enter as well as do quality control of the data entered. Data were abstracted directly from registers of TB patients who were initiated on TB treatment from 1 January 2013 to 31 December 2015. Furthermore, TB patients registered in the TB register in 2012 and died in 2013 were also included in the data extraction. (Line 147 to 152 Page 8). The data entry clerk was under strict instruction to enter data anonymously from the TB registers onto the database. No identifiers were extracted from the TB registers. It should be noted that this data were extracted in 2016 of patients who received service for TB treatment almost 3 years previously. As a result it was not possible to get patient consent as these were historical records and patients had been out of the system for 3 years already by the time the study was being conducted, so we had to seek waiver of consent to use the data as we believed that we could generate informative data that would shape policy and treatment guidelines in the management of TB at a time when expanding access to TB/HIV collaborative treatment was actively being rolled out by the department of

health as a successful model of clinical care to minimise poor outcomes. Ethical approval was granted by the Monash University Human Research Ethics Committee (approval number: CF16/2803- 2014001548). Permission to conduct the study at Elim Primary Health Care Clinic was obtained from the Director of in Ugu Health District, KwaZulu Natal.

Comment 3. Thank you for stating the following financial disclosure:

Response: *N/A*

At this time, please address the following queries:

a) Please clarify the sources of funding (financial or material support) for your study. List the grants or organizations that supported your study, including funding received from your institution. N/A
b) State what role the funders took in the study. If the funders had no role in your study, please state: "The funders had no role in study design, data collection and analysis, decision to publish, or preparation of the manuscript." N/A
c) If any authors received a salary from any of your funders, please state which authors and which funders. N/A
d) If you did not receive any funding for this study, please state: "The authors received no specific funding for this work."

Please include your amended statements within your cover letter; we will change the online submission form on your behalf.

Response: *In track change document on page 26, Line 492. Thanks, we state this: "Financial Disclosure: The author(s) received no specific funding for this work".*

Comment 4. In your Data Availability statement, you have not specified where the minimal data set underlying the results described in your manuscript can be found. PLOS defines a study's minimal data set as the underlying data used to reach the conclusions drawn in the manuscript and any additional data required to replicate the reported study findings in their entirety. All PLOS journals require that the minimal data set be made fully available. For more information about our data policy, please see http://journals.plos.org/plosone/s/data-availability.

Upon re-submitting your revised manuscript, please upload your study's minimal underlying data set as either Supporting Information files or to a stable, public repository and include the relevant URLs, DOIs, or accession numbers within your revised cover letter. For a list of acceptable repositories, please see http://journals.plos.org/plosone/s/data-availability#loc-recommended-repositories. Any potentially identifying patient information must be fully anonymized.

Important: If there are ethical or legal restrictions to sharing your data publicly, please explain these restrictions in detail. Please see our guidelines for more

information on what we consider unacceptable restrictions to publicly sharing data: http://journals.plos.org/plosone/s/data-availability#loc-unacceptable-data-access-restrictions. Note that it is not acceptable for the authors to be the sole named individuals responsible for ensuring data access.

We will update your Data Availability statement to reflect the information you provide in your cover letter.

Response: *In track change document on page 23, lines 423-424. Thanks, Dataset is available upon request by submitting the request form to the corresponding author (email: pnyasulu@sun.ac.za).*

Comment 5. We note that Figure 1 in your submission contain map images which may be copyrighted. All PLOS content is published under the Creative Commons Attribution License (CC BY 4.0), which means that the manuscript, images, and Supporting Information files will be freely available online, and any third party is permitted to access, download, copy, distribute, and use these materials in any way, even commercially, with proper attribution. For these reasons, we cannot publish previously copyrighted maps or satellite images created using proprietary data, such as Google software (Google Maps, Street View, and Earth). For more information, see our copyright guidelines: http://journals.plos.org/plosone/s/licenses-and-copyright.

We require you to either (1) present written permission from the copyright holder to publish these figures specifically under the CC BY 4.0 license, or (2) remove the figures from your submission:

a. You may seek permission from the original copyright holder of Figure 1 to publish the content specifically under the CC BY 4.0 license.

We recommend that you contact the original copyright holder with the Content Permission Form (http://journals.plos.org/plosone/s/file?id=7c09/content-permission-form.pdf) and the following text:
“I request permission for the open-access journal PLOS ONE to publish XXX under the Creative Commons Attribution License (CCAL) CC BY 4.0 (http://creativecommons.org/licenses/by/4.0/). Please be aware that this license allows unrestricted use and distribution, even commercially, by third parties. Please reply and provide explicit written permission to publish XXX under a CC BY license and complete the attached form.”

Please upload the completed Content Permission Form or other proof of granted permissions as an "Other" file with your submission.

In the figure caption of the copyrighted figure, please include the following text:
“Reprinted from [ref] under a CC BY license, with permission from [name of publisher], original copyright [original copyright year].”

b. If you are unable to obtain permission from the original copyright holder to publish these figures under the CC BY 4.0 license or if the copyright holder's requirements are incompatible with the CC BY 4.0 license, please either i) remove the figure or ii) supply a replacement figure that complies with the CC BY 4.0 license. Please check copyright information on all replacement figures and update the figure caption with source information. If applicable, please specify in the figure caption text when a figure is similar but not identical to the original image and is therefore for illustrative purposes only.
The following resources for replacing copyrighted map figures may be helpful:

USGS National Map Viewer (public domain): http://viewer.nationalmap.gov/viewer/
The Gateway to Astronaut Photography of Earth (public domain): http://eol.jsc.nasa.gov/sseop/clickmap/
Maps at the CIA (public domain): https://www.cia.gov/library/publications/the-world-factbook/index.html and https://www.cia.gov/library/publications/cia-maps-publications/index.html
NASA Earth Observatory (public domain): http://earthobservatory.nasa.gov/
Landsat: http://landsat.visibleearth.nasa.gov/
USGS EROS (Earth Resources Observatory and Science (EROS) Centre) (public domain): http://eros.usgs.gov/#
Natural Earth (public domain): http://www.naturalearthdata.com/

Response: *Thanks for this comment. The map was created using a shapefile from the Africa map library software, which was then imported into the Quantum Geographic information system* **(***QGIS) software for georeferencing of the Ugu district.*

Comment 6. Please upload a copy of Supporting Information S1 Dataset which you refer to in your text on page 18.

Response: *In track change document on page 21 and lines 399. Thanks, we have uploaded the data analysis commands.*

Reviewer #1

Comment 1: although this statement maybe correct, it cannot be a conclusion at the level of the abstract as the reader has no comparator.

Response: *In track change document on page 3 and lines 46 to 49. The conclusion is now: "when compared HIV positive and HIV negative status, antiretroviral treatment had no effect on the likelihood of TB cure in rural primary care. The TB mortality rate in HIV positive patients, on the other hand, was higher than in HIV negative patients".*

Comment 2: based on this what is your recommendation.

Response: *In track change document on page 3 and lines 49 to 50. We recommend this: "Various HIV/TB indicators should be reviewed, and gaps filled in order to achieve the "End TB strategy" in rural South Africa".*

Comment 3: comma

Response: *In track change document on page 4 and line 64, the comma has been inserted.*

Comment 4: I suggest that paragraph 2 is integrated at this point to improve the flow and readability of the manuscript.

Response: *In track change document on page 4 and lines 65-71. Thanks, paragraph 2 has been integrated as suggested.*

Comment 5: I think that this sentence is redundant information.

Response: *In track change document on page 4 and lines 75-77. Thanks, this sentence has been written more clearly in the background.*

Comment 6: There are two concepts being discussed in this sentence- decrease in mortality and increased incidence. however, the sentence needs multiple readings to make sense. therefore, I suggest that the sentence is separated.

Response: *In track change document on page 4 and lines 63-66. We have separated the two concepts. It now reads: "The increase in incidence is also attributed to the development of multidrug resistant (MDR) and extremely drug resistant (XDR) strains of Mycobacterium tuberculosis [34]. Both MDR/XDR-TB are the causes of high TB mortality [4]".*

Comment 7: This sentence appears out of sync as the previous discussion only relate to mortality and this sentence intends on providing reason for increased risk for TB.

Response: *The sentence has been deleted and removed.*

Comment 8: Which countries as the authors quoting facility based postmortem findings.

Response: *In track change document on pages 4-5 and lines 75-77. Thanks, we have listed the countries reporting post-mortem TB rate. It now reads: "such as South Africa*

[9-11], Botswana [11, 12], Zimbabwe [11, 13, 14], Mozambique [11, 15], Uganda [11, 16] and Kenya [11, 17]".

Comment 9: I am sure that the WHO global report on TB provides data in resource limited countries.

Response: *In track change document on page 5 and lines 77. Thanks, we have provided TB data in Africa. "In contrast, the WHO reported 16% of HIV/TB related death in Africa [1]".*

Comment 10: please update this data as later information is available.

Response: *In track change document on pages 5 and lines 80-81. Thanks, this data has been updated. "TB accounted for the third highest number of deaths in 2018 (6 %; n = 454 014)"*

Comment 11: specific data for Ugu district

Response: *In track change document on page 5 and lines 87-88. "Ugu district reported 60.5% of TB/HIV co-infection rate [25]".*

Comment 12: provide information on South Africa antiretroviral treatment programme in terms of number of patients on treatment and accessibility to treatment.

Response: *In track change document on pages 6 and lines 89-90. Thanks, we have reported this: "*According to recent data*, 90% of people are aware of their HIV status of these 68% are on antiretroviral therapy (ART) of which 87% are virally suppressed in SA [26]".*

Comment 13: this paragraph is out of sync in building the case. it may be more suited to a discussion

Response: *In track change document on pages 19-20, lines 360-367. Thanks, this paragraph has been moved to the discussion section.*

Comment 14: with a view to?

Response: *In track change document on page 7 and lines 141. Thanks, we state as follows: "with a view to Ugu district".*

Comment 15: the author needs to separate study design, population and study setting

Response: *In track change document on pages 8-9 and lines 122-142.Thanks, we have separated the study design, population and study setting. It is now reads:*

"Study design

This retrospective cohort study of TB patients initiated on TB treatment was conducted from 1 January 2013 to 31 December 2015.

Study population

We included all patients diagnosed with TB irrespective of their age and HIV status in the study. Further, we registered patients that were in the TB register in 2012 and died in 2013. We also included as well as those that died or survived during the treatment period. Patients with unknown outcome were from the study or those with incomplete record were excluded. Basic demographic information including the age, gender, co-morbidities, tobacco Use, alcohol Use, substance use and duration on treatment were collected.

Study setting

The Ugu district is located in the rural KwaZulu-Natal province (Figure 1). Ugu district has a population of 733 228 people [37]. During the study period, Ugu district had the highest HIV prevalence and TB incidence of any district in KZN, 41.7% and 1096 per 100,000 people, respectively [37]. In terms of infectious TB (pulmonary smear-positive), Ugu ranks 12th, with 325 cases per 100,000 people, which is higher than the country's average of 208 cases per 100,000 people [37]. Elim clinic, a primary health care facility was selected based on convenience, the study goal and the availability of information on HIV and TB infections".

Comment 16: this is about uGu- what about the specific area of your study- what is catchment population, what is outpatient headcount? what proportion or incidence of TB per year over the study period?

Response: *In track change document on pages 7 and lines 136-142. Thanks, we have stated the following:* ***"****Ugu district has a population of 733 228 people [37]. During the study period, Ugu district had the highest HIV prevalence and TB incidence of any district in KZN, 41.7% and 1096 per 100,000 people, respectively [37]. In terms of infectious TB (pulmonary smear-positive), Ugu ranks 12th, with 325 cases per 100,000 people, which is higher than the country's average of 208 cases per 100,000 people [37]. Elim clinic, a primary health care facility was selected based on convenience, the study goal and the availability of information on HIV and TB infections".*

Comment 17: was provincial authorization obtained? I am confused by the term Director of Health as no such position exists in the organogram.

Response: Thanks for this comment. Authorisation to access clinic records was granted by the Ugu Health District Manager. Apologies for the incorrect use of word 'Director'.

Comment 18: it would be good to also provide the socio-demographic profile of patients first before the HIV status of patients.

Response: *In track change document on page 12and line 240. Thanks, before the HIV status table (Table 2), we have provided a socio-demographic table (Table 1).*

Comment 19: p values determination- and if any of the information is statistically significant

Response: *In track change document on page 15, table 3, line 277. Thanks, p-values have been provided. Duration on treatment and age-group were significant predictors of cure.*

Comment 20: were the values statistically significant?

Response: *In track change document on page 15, line 277. "The 95% CI shows that the values obtained were statistically significant".*

Comment 21: to evaluate TB outcomes in HIV positive patients in rural primary healthcare in South Africa

Response: *In track change document on page 17, lines 300. Thanks, it now reads: "This study evaluated the effect of HIV status and antiretroviral treatment on the treatment outcome of TB patients in a primary healthcare facility in rural South Africa".*

Comment 22: what about findings from your analytical study?

Response: *In track change document on page 17, lines 312-317. We have reported the findings from the analytical study as follows: "Longer treatment periods were associated with a lower risk of death in both the bivariate and covariate log-binomial regression models. Furthermore, in bivariate and covariate analysis, younger ages had a lower likelihood of being cured than older ages. However, HIV positive status of a TB patient had no effect on the likelihood of TB cure when compared to HIV negative status of a TB patient".*

Comment 23: please clarify this- is this initiation of treatment or treatment duration

Response: *In track change document on page 17, lines 315-317. It now reads "However, HIV positive status of a TB patient had no effect on the likelihood of TB cure when compared to HIV negative status of a TB patient".*

Comment 24: how does this study relate to your findings. what is the plausible explanation for the similarities and differences?

Response: *In track change document on page 18, lines 329-340. Thanks, the plausible explanation for the similarities and differences is stated as follows: "The*

similarities and differences between studies could be explained by ART uptake percentage and TB diagnosis. Following a review of these studies, the findings of our study was consistent with the study conducted in Kenya, owing to the ongoing scale-up and uptake of ART programs in both South Africa and Kenya. Table 2 of our study revealed an ART uptake rate of 89.9%, compared to 61% in Kenya [42]. In contrast, 3.7% of ART uptake was recorded in Ghana [40] and 16% patients with extrapulmonary TB did not receive ART in Tanzania [41]. Aside from that, our study found 70.0% of patients to have pulmonary TB, compared to 36.9% in a Tanzanian study [41]. In fact, pulmonary TB is easier to diagnose than other types of TB. Previous data shows that "Extrapulmonary TB is associated with poor TB outcome in HIV-infected people [43]".

Comment 25: is better adherence to treatment the only plausible explanation? if you review the data. It is noticed that age > 50 years also had a very high likelihood of mortality and therefore, i question your statement provided.

Response: *In track change document on page 19, lines 349-353. Thanks, we have provided the following statement:" Another study has shown that older ages are more likely to develop extra-pulmonary and atypical forms of TB disease that are often harder to diagnose than conventional sputum smear-positive pulmonary tuberculosis [45]. Extra pulmonary and smear negative PTB were associated with high TB mortality [43, 46, 47]."*

Comment 26: what is the plausible explanation for tobacco and TB treatment outcomes?

Response: *In track change document on page 19, lines 362-366. The discussion part is improved as follows: "Smoking tobacco affects both innate and adaptive immunity, weakening the immunological defensive system in humans [50]. This appears to be the reason why smokers are at a higher risk of developing extra-pulmonary tuberculosis [51]. Apart from this, smoking tobacco increases the risk of mycobacterium TB infection as well as the development of tuberculosis in infected individuals [52]".*

Comment 27: please discuss the study limitations

Response: *In track change document on page 20, lines 381-388. We have included the study strengths and limitations. It now read: "The strength of our study is our sample size which was representative of the study population thereby minimizing selection bias. This is substantial to estimate HIV/TB outcomes in rural settings. Our study has several limitations that could lead to underestimation HIV/TB outcomes. Retrospective cohort design uses records that have already been collected and we did not obtain the information on treatment completed outcome, HIV viral load and CD4 cells. As the fact, we were unable to accurately link all our patients to the various outcomes. It is also possible that some of the patients included in our study misclassified in the current analysis".*

Comment 28: this is a repetition of results without adequate considering for the overall aim and why the study was done?

Response: *In track change document on page 21, lines 392-395, 400-404. Thanks, we have improved the conclusion as follows: "In conclusion, HIV positive status and antiretroviral treatment had no effect on the likelihood of TB cure in rural primary care when compared to HIV negative patients. However, the TB mortality rate in HIV positive patients was higher than in HIV negative patients". And further recommendation: "TB mortality in rural SA. Furthermore, the TB success rate in rural SA may be lower than the WHO target. This study could have a significant impact on the HIV/TB program in rural SA. To achieve the End TB strategy in rural SA, various HIV/TB indicators should be reviewed, and gaps filled".*

Reviewer #2:

Comment 1: The abstract is well written. It contains the main findings and the conclusion of the study. The background is comprehensive and well written. Although, it does not reflect on the decreasing TB cases that South Africa is experiencing.

Response: *In track change document on page 5-6, lines 103-108. Thanks, we have improved the background as follows:" TB incidence and mortality are declining in SA [7]. Data from a well-characterized rural SA population with high HIV prevalence and TB incidence demonstrated considerable spatial heterogeneity in people with recently-diagnosed TB and has shown that every percentage increase in ART coverage was associated with a 2% decrease in the odds of recently-diagnosed TB [23]".*

Comment 2: The methodology is clear, well defined. The paper does not clearly define treatment cure, treatment completed.

Response: *In track change document on pages 8, lines 160-167. We have referred to the WHO definitions.* ***Cured:*** *A pulmonary TB patient with bacteriologically confirmed TB at the beginning of treatment who was smear or culture negative in the last month of treatment and on at least one previous occasion.* ***Treatment completed:*** *A TB patient who completed treatment without evidence of failure but with no record to show that sputum smear or culture results in the last month of treatment and on at least one previous occasion were negative either because tests were not done or because results are unavailable [37].*

Comment 3: The researcher used the outcome allocated by the facility. I am not sure whether these outcomes were checked. Sometimes patients who fulfil the cure criteria are captured as completed. It is also important to highlight that the outcomes are allocated mainly on the basis of negative TB smear microscopy. TB microscopy has a low sensitivity. TB culture is not done routinely in susceptible TB patients.

Response: *In track change document on page 20, lines 384-388. Thanks, we have addressed this issue in the study weaknesses. As a retrospective cohort study, we conducted the records review of the patients. As a matter of fact, we were unable to accurately link all of our patients to the various outcomes due to missing data. It is also possible that some of the patients included in our study might have been misclassified in the current analysis.*

Comment 5: My other issue is the choice of comparing cure or death. TB programme targets are based on treatment success rate (Cure and Completion). I agree that it is better to have a higher cure rate although for several reasons I have noted over the years the final sputum is often not collected hence the outcome will be "treatment completed".

Response: *Thanks for the observation, as previously stated, retrospective cohort design used records that have already been collected and we did not obtain the information on treatment completed from the records.*

Comment 6: Also, Table 1 indicates that 30 % of the cohort had a negative smear microscopy. Such patients may not have a "cure" outcome. Favorable outcomes include cure and completion rate. I am not sure what is the motivation for having cure, death, and other outcomes. It would have been useful to briefly explain why are there more treatment completion than treatment cure in this facility.

Response: *In track change document on page 9, lines 160-163. Thanks, “cured” is defined as a pulmonary TB patient with bacteriologically confirmed TB at the beginning of treatment in an individual who was smear or culture negative in the last month of treatment and on at least one previous occasion. So, people may complete TB treatment without necessarily being cured. In this study we used these outcomes because they are key TB Program indicators as defined by the National TB Control Program. The focus of our study was to assess the effect of HIV status and antiretroviral treatment on TB treatment outcomes including cure. Our data has shown that HIV status and antiretroviral treatment did not have effect on TB cure but on TB mortality. We suggest that further studies are warranted to help explain low TB cure in this setting.*

Comment 7: Data analysis is very clear except the issue of separating cure and completion. On page 11 of the manuscript, the Authors stated that among tobacco users the cure rate was for TB was 60 % and it was only 37 % among the non-tobacco users. In the conclusion, it is stated that the use of tobacco appeared to decrease the TB cure. It is also said that the cure rate was below district and provincial targets. To the best of my knowledge, there are no separate targets between cure and completion. Targets are based on Treatment Success Rate.

Response: *Thanks for the observations, In track change document on page 19-20, Table 4. We separated cure and completion because they are two separate National TB Control Program treatment outcomes. The overall cure rate was 39.13% which falls below district and provincial targets based on South African District Health Barometer. Treatment Success Rate is the sum of cured and treatment completion rates.*
