# Supplementary material for: Effect of HIV status and antiretroviral treatment on treatment outcomes of tuberculosis patients in a rural primary healthcare clinic in South Africa
Source: PLoS One. 2022 Oct 12;17(10):e0274549. doi: 10.1371/journal.pone.0274549 (PMC9555649; doi:10.1371/journal.pone.0274549)
Supplement: Attachment — Submitted filename: PONE_Reviewers responses_July 16_2022.docx [file pone.0274549.s005.docx]

**Reviewers’ responses to reviewers**

**Title: Effect of HIV status and antiretroviral treatment on treatment outcomes of tuberculosis patients in a rural primary healthcare clinic in South Africa**

**Journal Requirements:**

**Comment 1:** Please review your reference list to ensure that it is complete and correct. If you have cited papers that have been retracted, please include the rationale for doing so in the manuscript text or remove these references and replace them with relevant current references. Any changes to the reference list should be mentioned in the rebuttal letter that accompanies your revised manuscript. If you need to cite a retracted article, indicate the article’s retracted status in the References list and include a citation and full reference for the retraction notice.

***Response 1:*** *Thank you for this guidance. In the tracking manuscript, the reference 10, 12, 14, 16, 17, 30, and 31 have been revised according to PLOS One submission guideline.*

**Reviewer #1:**

**Comment 1:** The authors have addressed most of the substantive points of the previous review. I have highlighted certain areas on the re-submission that need to be looked at again. However, the authors have data from more than 5 years ago and do not provide a critical discussion of how this old data still stands currently with the growth and changes in the program. Is it not likely that a greater saturation in terms of ART has improved TB outcomes or there is a worsening due to COVID-19.

***Response 1:*** *Thanks for this valuable comment. In the tracking document lines 382-396, the following has been highlighted: “Coronavirus disease 2019 (COVID-19) has a significant impact on TB services in addition to the previous TB challenges faced in rural areas in SA. A study conducted in a rural area in SA discovered a significant drop in total TB treatment enrolment from April to December 2020 when compared to April to December 2019, with the largest drop occurring in July 2020 (–26%) [54]. Data shows a decrease across all TB indicators in April 2020, the month of the first COVID-19 lockdown, with positive TB tests experiencing the greatest decrease (–33%) [54]. TB testing and diagnosis decreased by 50% and 33%, respectively, during the COVID-19 lockdown in SA [55, 56]. Furthermore, the National Health Laboratory Service (NHLS) reported that the number of GeneXpert tests performed decreased by 26% between 2019 and 2020, and the proportion of positive tests decreased by 18% in SA [57]. Given that the GeneXpert is a key diagnostic test for TB in HIV-positive people, the TB mortality rate in HIV-positive patients may be higher in rural areas during the COVID-19 pandemic. In addition to the findings of this study, COVID-19 may jeopardize the End TB strategy in rural area in SA.”.*

**Reviewer #2:**

**Comment 1:** on what premise is this conclusion derived to be presented in the abstract

***Response 1:*** *Thanks for the observation. In tracking manuscript line 46-49, we have changed the conclusion as follows:* *“When compared between HIV status, HIV positive TB patients were more likely to have unsuccessful treatment outcome in rural primary care”. Antiretroviral treatment seems to have had no effect on the likelihood of TB treatment success in rural primary care.* *The TB mortality rate in HIV positive patients, on the other hand, was higher than in HIV negative patients emphasizing the need for enhanced integrated management of HIV/TB in rural South Africa through active screening of TB among* HIV positive individuals and early access to ART among HIV positive TB cases.

**Comment 2:** these results have not been presented in abstract

***Response 2:*** *Thanks for highlighting this. In the tracking manuscript line 42-43, “**Furthermore, HIV positive patients had a higher mortality rate (9.67%) than HIV negative patients (2.91%)”.*

**Comment 3:** at this point this has no bearing with the results in abstract or link to introduction

***Response 3:*** *Thanks for this comment, the conclusion has been revised based on above comment 1 and 2.*

**Comment 4:** the term evaluate has a deeper meaning than just an epidemiological study as conducted by the authors

***Response 4:*** *Thanks. In the tracking manuscript line 118, It reads: “Therefore, this retrospective study has been undertaken to determine….”*

**Comment 5:** geo spatial map of Ugu and the key TB indicators in comparison to other districts in the Province will be beneficial for external readers to picture the burden in Ugu

***Response 5:*** *Thanks for the guidance. Figure 1 has been designed according to this recommendation.*

**Comment 6:** out of sync

***Response 6:*** *Thanks for this observation. In the tracking manuscript, line 145, the title has been aligned.*

**Comment 7:** based on what you have provided you have not evaluated you have only determined factors associated with

***Response 7:*** *Thanks for the observation. In the tracking manuscript, line 308, we have replaced “evaluated” to “determined”.*

**Comment 8:** please clarify what HIV status is

***Response 8:*** *Thanks for this advice. Line 326, we have specified as follows: “HIV positive status”.*

**Comment 9:** what is recent

***Response 9:*** *Thanks for this point seeing clarity. Line 371, “recent” has been removed from this sentence with appropriate edit.*

**Reviewer #3:**

**Comment 1:** Line 126: would it have been helpful to distinguish pediatric from adult participants given likely baseline differences in HIV and TB risk?

***Response 1****: Thanks for this observation. In the tracking document line 223-224: “Among them, only 13% (66/506) were children”. We do not believe that removing it will change the outcome.*

**Comment 2:** Line 182: what proportion of data were missing and how were missing data accounted for?

***Response 2:*** *Thanks for this question. In the tracking document line 409-415:* *“Additionally, 5.5% of the HIV status was missing, which might have hampered our understanding of the effect on HIV on mortality among people with TB. To overcome missing data challenges, we used complete case (or available case) analysis or listwise deletion approach, hence include in the final analysis only cases with complete data available.*

**Comment 3:** Was HIV status confirmed based on seropositivity using chart abstraction? If based on self-report, may result in measurement bias, possibly underestimating true HIV prevalence.

***Response 3:*** *Thanks for this comment. As part of a retrospective study design, HIV status was confirmed using medical records available to ascertain objectiveness.*

**Comment 4:** Use of TB cure definition using smear negativity may miss cases that are smear negative and go on to become culture positive. Use of completed TB definition may include persons who were cured following treatment.

***Response 4:*** *Thanks for this comment. Given that the study outcome includes “cured” and “treatment completion”, we have added the following definition: “Treatment success includes the sum of cured and treatment completed [38]” (Line 168-169). “Cured” has been replaced by “treatment success” in the following line 49, 168, 172, 173, 226, 228, 231, 233, 232, 233, 235-238, 280, 286, 347, 348, and 422.*

**Comment 5:** Based on baseline TB regimens, it appears none of the patients were being treated for multidrug resistant TB (which is prevalent in South Africa); this may limit generalizability/external validity.

***Response 5:*** *Thank for this valuable comment. Line 409-411.* *We have added the following: “Other study limitations included the fact that no patients were diagnosed nor treated for MDR-TB at this clinic, which may limit the study's generalizability”.*

**Comment 6:** Was matching on covariates between HIV positive and HIV negative participants considered?

***Response 6:*** *Thanks for this question. Since HIV status was an important covariate in the statistical modelling, we didnot perform matching of variables.*
